# Anion-Induced Reversible Actuation of Squaramide-Crosslinked Polymer Gels

**DOI:** 10.1021/acsami.2c11136

**Published:** 2022-09-13

**Authors:** Stefan Mommer, Sander J. Wezenberg

**Affiliations:** Leiden Institute of Chemistry, Leiden University, Einsteinweg 55, 2333 CC Leiden, The Netherlands

**Keywords:** anion binding, squaramide, polymer gels, soft actuators, smart materials

## Abstract

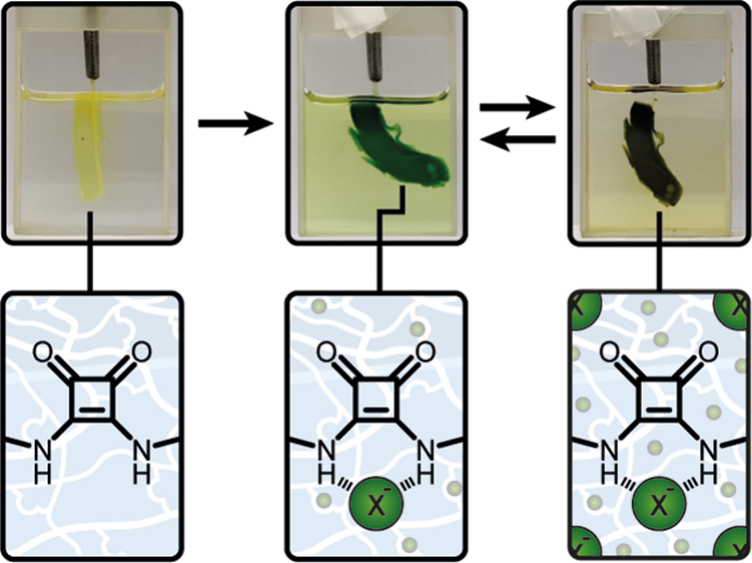

Supramolecular anion binding to squaramide crosslinkers in poly(*N*,*N*-dimethylacrylamide) gel networks enhances swelling and allows reversible chemically driven actuation. The volume swelling ratio of the gels is shown to depend on both the type of anion and its concentration. ^1^H NMR and UV–vis titrations with the squaramide crosslinkers reveal a relationship between anion binding affinity and the concentration-dependent swelling behavior. Gel swelling is shown to be reversible, and by embedding a solid support into rod-shaped gels, soft actuators are fabricated that undergo forward and backward bending motion in response to changing anion concentration. The swelling and bending process, which is accompanied by intense green coloration of the gel, is achieved by using only low amounts of crosslinker. This macroscopic actuation achieved by anion binding to specific molecular entities in the polymer network will open new opportunities in the field of chemically responsive materials.

## Introduction

The ability of living organisms to adapt their shape and color to the surrounding environment has been a constant source of inspiration in the design of smart materials.^1−4^ Polymeric gels are well suited in this regard owing to their flexible and penetrable nature. Indeed, soft actuators made from stimuli-responsive polymer gels have emerged as a thriving research field, with promising applications in electronics, healthcare, and robotics.^5−15^ Actuation of these gels is generally induced by stimuli such as heat, light, or an electrical or magnetic field.^16−20^ In particular for medical applications, however, chemically responsive actuators would be beneficial, because they could act upon internal stimuli in the body where external stimuli can be hard to apply. Yet, successful examples of mechanical actuation driven by changes in the chemical environment (i.e., chemomechanical actuation) are limited and typically based on changes in pH value, humidity,^5,6,9−15^ or—more rarely—site-specific interaction with metal cations.^7,8,21−24^

Anions and anionic substances are omnipresent in nature and fulfill important roles in various chemical and biological processes. A large number of artificial anion receptors have therefore been developed,^25−27^ which have proven useful in applications such as analyte sensing,^28,29^ wastewater extraction,^30^ and transmembrane transport.^31−33^ Additionally, anions have been used to alter the properties of low-molecular-weight supramolecular gels.^34−36^ Their binding to certain gelators can result in disruption of an intermolecular hydrogen bonding network, leading to an irreversible gel–sol transition. Yet, while complex and programmable shape deformation of polymer gels has been achieved by various physical and chemical stimuli,^11,14^ to our best knowledge, reversible actuation of (polymeric) soft materials through binding of anionic species has not been demonstrated.

Some anion receptors have been attached to polymeric supports in order to improve sensing and extraction properties.^8,37^ For example, thiourea-functionalized polymers have been used as colorimetric sensors for acetate and bicarbonate,^38^ while urea-containing polymers have shown enhanced binding of phenylphosphonate.^39^ Interestingly, Flood and co-workers found that incorporation of aryl-triazole units into poly(methyl methacrylate) (PMMA) did not only enhance the chloride extraction capability but that formation of crosslinks by the anion also affected the polymer’s hydrodynamic radius.^40^ In addition, the group of Sessler observed that anion extraction using PMMA copolymers bearing calix[4]pyrrole or tetracationic macrocycles altered the polymer’s physical properties because of receptor–anion interactions.^41,42^ Despite these observations, efforts to gain control over macroscopic properties of polymer materials using anionic stimuli are scarce. So far, to the best of our knowledge, only the groups of Sada^43^ and Song^44^ reported anion-dependent swelling (and in the latter case also bending) of thiourea-containing polymer gels. However, no reversibility was demonstrated. We envisioned that the use of a stronger anion-binding motif would enhance swelling at low anion concentration, which, in addition to the previously described swelling decrease in the presence of excess amount of anion salt,^43,44^ could give rise to reversible behavior.

**Figure 1:**
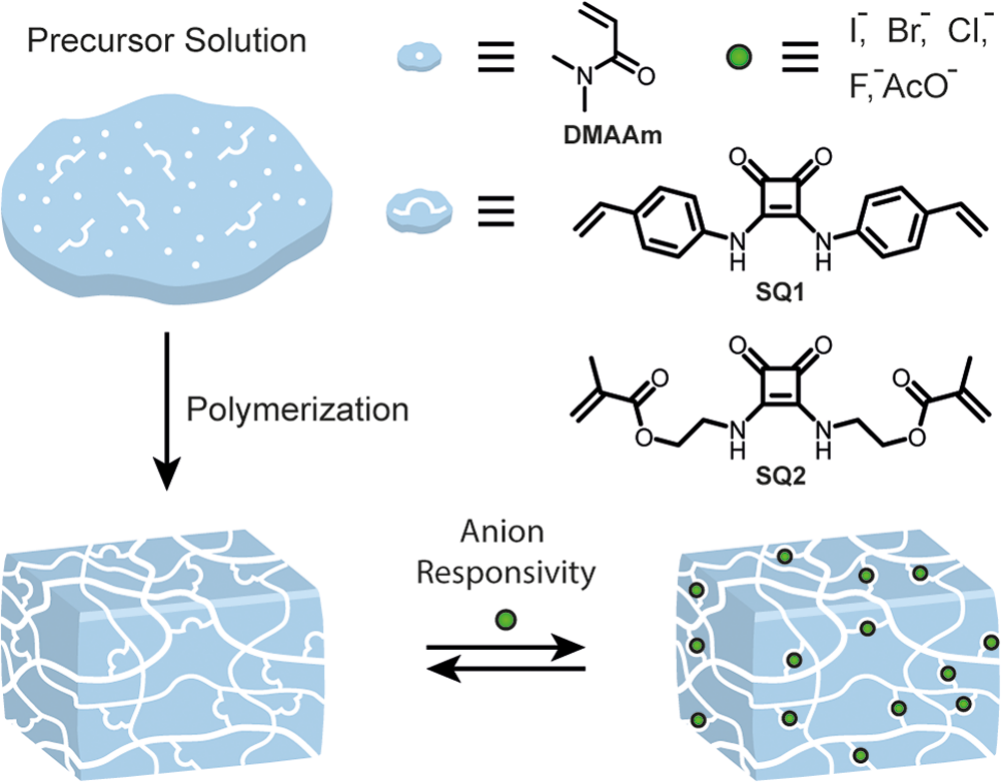
Preparation of anion-responsive polymeric gels using crosslinkers **SQ1** and **SQ2**.

Herein, we present the anion-mediated reversible swelling and actuation of polymer gels that contain squaramide crosslinkers **SQ1** and **SQ2** (Figure 1). Squaramide has superior anion binding affinity compared with thiourea.^45,46^ Significant swelling can be achieved with only 5 mol % of crosslinker, and the swelling volume is shown to depend on both the type and the concentration of the anion. Fabrication of the gel in a rectangular shape on a solid support affords a soft actuator, which undergoes reversible bending in response to changes in anion concentration. Furthermore, the anion-specific gel swelling and bending process is accompanied by a stark colorimetric change, adding sensing functionality to the polymer material.

## Results and Discussion

### Monomer Synthesis and Polymerization

The design of crosslinkers **SQ1** and **SQ2** was derived from well-established squaramide anion receptors (see Figure 1).^45,46^ The former, with two attached styryl units, making it susceptible to polymerization, was developed earlier by Manesiotis et al., who evaluated it in the extraction of phosphate and benzoate, as well as organo-arsenic compounds.^47,48^ By using a slightly modified procedure, that is, substitution of squaric acid diethyl ester with vinyl aniline in the presence of catalytic amounts of zinc triflate, **SQ1** was obtained in 97% yield (see Figures S1–S5). While electron-withdrawing aromatic substituents increase squaramide proton acidity and hence anion binding affinity,^45,46^ they will add to the rigidity of the crosslinker. For comparison, we therefore also synthesized the aliphatic derivative **SQ2**, which comprises two methacrylate groups, providing a higher degree of molecular flexibility. Because of increased electron density, this derivative should have lower anion binding affinity with respect to **SQ1**. The synthesis of **SQ2** was carried out in similar way as for **SQ1**, now using 2-aminoethyl methacrylate instead of vinyl aniline in the substitution reaction. Furthermore, as opposed to zinc triflate as catalyst, *N*,*N*-diisopropylethylamine was used in this case as the acid scavenger to give the desired compound in 48% yield (see Figure S6–S10).

To obtain the anion-responsive gel networks, free radical polymerization using *N*,*N*-dimethylacrylamide (DMAAm) as main monomer was chosen. In contrast to, for example, acrylamide or dimethylaminoethyl acrylate, DMAAm does not have NH protons that could engage in interactions with anions, thus allowing us to solely investigate the effect of anion binding to the squaramide crosslinkers. Polymerizations were carried out in small volumes (0.25 mL) with a 1.0 M concentration of the main DMAAm monomer (10 wt % in DMSO), 5 mol % of either the **SQ1** or **SQ2** crosslinker, and 1 mol % of thermoinitiator AIBN. The samples were placed in an oven to cure overnight at 60 °C resulting in small pellet-like gel specimens.

### Anion-Induced Swelling Behavior

To characterize the macroscopic behavior of the polymer networks, the obtained gel specimens were subjected to swelling experiments. In general, if a gel sample is immersed in excess solvent, the surrounding solution may exhibit an osmotic pressure on the network to balance the free energy of mixing (*F*_mix_), which is counteracted by the networks’ contractile elastic energy (*F*_el_).^49,50^ An influx of solvent molecules into the network will then lead to a volume expansion until the equilibrium swollen state (*F*_mix_ = −*F*_el_) is reached. It should be noted that in nonionic networks this volume expansion is normally independent of the concentration of salts being present. We hypothesized that, in our case, the squaramide crosslinkers would infuse the nonionic network with anion-specific swelling behavior, introducing a third energy contribution (*F*_ion_) to account for (*F*_mix_ + *F*_ion_ = −*F*_el_).^51,52^

**Figure 2:**
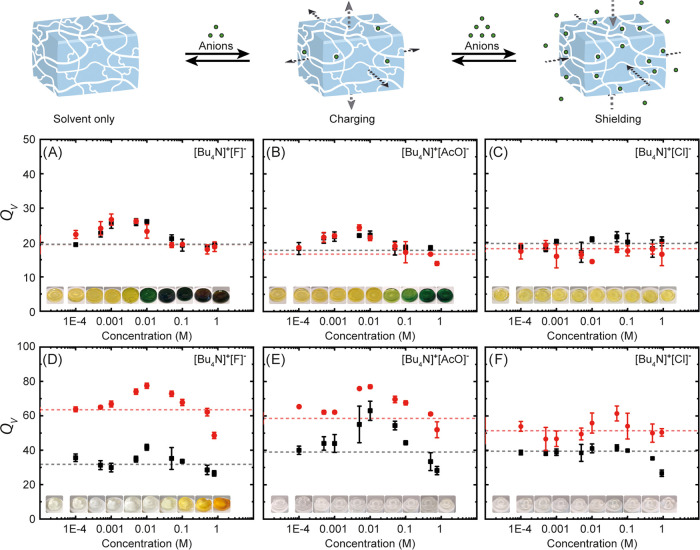
Volume swelling ratio vs anion concentration of **SQ1**-crosslinked gels swollen in (A) [Bu_4_N]^+^[F]^−^, (B) [Bu_4_N]^+^[AcO]^−^, and (C) [Bu_4_N]^+^[Cl]^−^ as well as **SQ2**-crosslinked gels swollen in (D) [Bu_4_N]^+^[F]^−^, (E) [Bu_4_N]^+^[AcO]^−^, and (F) [Bu_4_N]^+^[Cl]^−^. Data points were determined in triplicates and represent equilibrium swelling after 24 h (black squares) and 7 days (red spheres). Dashed lines show the volume swelling ratio in salt free solutions. Inset pictures represent sample specimens after swelling 24 h in the respective anion solution with increasing concentrations: 0, 0.1, 0.5, 1, 5, 10, 50, 100, 500, and ∼1000 mM.

Henceforth, the obtained gel specimens were immersed in DMSO solutions containing different concentrations of anions (F^–^, Cl^–^, Br^–^, I^–^, AcO^–^; tetrabutylammonium salt) for 24 h or 7 days. After this time, the volume swelling ratios (*Q*_*v*_) were calculated to assess the expansion of the gels (see Figure 2 and eqs S1–S2 as well as Figures S37–S51 and the Supporting Information for full details). Interestingly, the gels showed a distinct swelling pattern dependent on the concentration for each anion. For example, when **SQ1**-crosslinked gels were immersed in solutions of [Bu_4_N]^+^[F]^−^, a peak-shaped profile was obtained for the volume swelling ratio as a function of the concentration (Figure 2A). After 24 h, a maximum of *Q*_*v*_ = 26.1 was observed at around 0.01 M, and an increase of the immersion time to 7 days did not lead to higher values, suggesting that the equilibrium swelling degree was reached within the first 24 h. In solutions of [Bu_4_N]^+^[AcO]^−^, **SQ1**-crosslinked gels showed a very similar pattern (Figure 2B). Here, at around 0.05 M, a maximum of *Q*_*V*_ = 24.4 was reached after 24 h, and again, extension of the immersion time to 7 days gave a similar swelling profile. In addition to this anion concentration-dependent swelling, the gel specimens changed color from yellow to dark green at the higher salt concentrations (Figure 2A,B, inset pictures), which can be traced back to squaramide deprotonation.^47^ It is important to note that this color change occurs in a different concentration regime than the maximum swelling enhancement.

When **SQ1**-crosslinked gels were submerged in solutions of [Bu_4_N]^+^[Cl]^−^, [Bu_4_N]^+^[Br]^−^, and [Bu_4_N]^+^[I]^−^, nearly flat swelling profiles were obtained (Figure 2C and Figures S34–S35), which is most likely due to a weaker interaction of the anion with the crosslinker as compared to the experiments with [Bu_4_N]^+^[F]^−^ and [Bu_4_N]^+^[AcO]^−^ (*vide infra*). In this case, also no color change was observed, highlighting that the gels could be used in the colorimetric sensing and detection of the more basic F^–^ and AcO^–^ anions. Experiments carried out with gels containing crosslinker **SQ2**, exhibiting a higher degree of molecular flexibility, showed similar peak-shaped swelling profiles. Now, after 24 h, maxima of *Q*_*V*_ = 41.7 and *Q*_*V*_ = 63.0 were obtained around 0.01 M for [Bu_4_N]^+^[F]^−^ and [Bu_4_N]^+^[AcO]^−^, respectively (Figure 2D,E).

In this case, the maxima increased to *Q*_*V*_ = 77.5 for [Bu_4_N]^+^[F]^−^ and *Q*_*V*_ = 77.0 [Bu_4_N]^+^[AcO]^−^ upon prolonging the immersion time to 7 days. These values are well above the volume swelling ratios obtained with the salt free solutions (*Q*_*V*_ = 40.3 and 58.6, respectively). In [Bu_4_N]^+^[Cl]^−^ solution, as was also observed for the gels containing **SQ1**, these **SQ2**-crosslinked gels displayed a flat swelling profile after 24 h and very minor concentration dependency after 7 days (Figure 2F), again indicative of a lower binding affinity. In contrast to **SQ1**-crosslinked gels, colorimetric changes were only observed for [Bu_4_N]^+^[F]^−^, which is in line with the less acidic NH protons of aliphatic squaramides with respect to aromatic ones, because they are less prone to deprotonation.^53^ Importantly, when commercially available ethylene glycol dimethacrylate (EGDMA) was used as the crosslinker, which does not bind anions, no concentration-dependent swelling was observed (Figure S36). This control experiment confirms our expectation that anion binding is crucial to enhance swelling of the squaramide-crosslinked gels.

The anion-specific and concentration-dependent swelling is thus explained by the adsorption of anions, which is facilitated by the squaramide crosslinkers (Figure 2). In brief, when immersed in salt free solutions, the gels expand to a certain degree,^54^ behaving like ordinary nonionic networks. However, in the [Bu_4_N]^+^[F]^−^ and [Bu_4_N]^+^[AcO]^−^ solutions, the polymer network becomes ionically “charged” owing to the presence of anion-binding sites. The enhanced swelling in this case, beyond the volume swelling ratio obtained using the salt free solution, can be ascribed to charge repulsion between polymer chains. At higher salt concentrations, however, the amounts of anions bound to the polymer (note that only 5 mol % of crosslinker is incorporated) becomes negligible as compared to the high quantities of salt being present inside and outside of the network. As a result, the charges close to the polymer chains become increasingly shielded by the surplus of salt, and the gels do not show enhanced swelling anymore, in line with the behavior of polyelectrolytes.^51,55^

Oscillatory rheological measurements were conducted to confirm the anion-induced swelling via appropriate changes in the stiffness of the gels. It is well-known that the degree of swelling directly correlates with the viscoelastic properties and, thus, storage and loss moduli of the gels. As the use of [Bu_4_N]^+^[F]^−^ gave large responses, samples of **SQ1**- and **SQ2**-crosslinked gels were each immersed for 24 h in 0.01 and 0.8 M solutions of this salt, as well as a salt free solution, after which oscillatory frequency sweeps were measured. For **SQ1**-crosslinked gels, the storage modulus (a measure for the stiffness and energy that can be stored within the network) for a gel immersed in 0.8 M [Bu_4_N]^+^[F]^−^ was determined as 390 Pa (Figure S52). This value is similar to that measured in salt free solution (380 Pa), which is in agreement with the similar volume swelling ratios (see Figure 2). For the gel swollen in the 0.01 M solution, however, the storage modulus decreased to 247 Pa, as an illustration of the larger swelling degree. Similar observations were made with **SQ2**-crosslinked gels (Figure S53). Here, the storage modulus was 136 Pa for the gel that was immersed in the 0.8 M salt solution and 93 Pa for the one in the salt free solution, while for the 0.01 M salt solution the value decreased to 63 Pa. These rheological measurements are thus in agreement with the volume swelling experiments described above. Furthermore, all measurements showed frequency-independent behavior, which is expected for covalently crosslinked polymer networks.

### Anion Binding Studies

To elucidate the influence of crosslinker–anion binding on the swelling behavior, ^1^H NMR and UV–vis spectroscopic titrations were carried out using **SQ1** and **SQ2** monomers. For ^1^H NMR titrations, a solvent mixture of DMSO-*d*_6_ and 0.5% H_2_O was used, and with HypNMR,^56^ the obtained ^1^H NMR chemical shift data was fitted to a 1:1 binding model (Figures S11–S20), in accordance with what has been reported for squaramide receptors.^57,58^ For both **SQ1** and **SQ2**, addition of [Bu_4_N]^+^[I]^−^ did not produce any noticeable chemical shift changes, suggesting that neither of them bound iodide (Figures S11 and S16). Where also the titration of **SQ2** with [Bu_4_N]^+^[Br]^−^ did not lead to noteworthy changes in chemical shifts, addition of this salt to **SQ1** produced a downfield shift of the squaramide NH signal, indicative of bromide binding (Figures S12, S17, and S21). Addition of [Bu_4_N]^+^[Cl]^−^ to **SQ1** and **SQ2** showed similar downfield shifting of the NH signal, and analysis of the titration data revealed weak to moderate binding (see Table 1 and Figures S13, S18, and S22–S23). The largest downfield shift was observed for the titration of **SQ2** with [Bu_4_N]^+^[AcO]^−^, and fitting of the data revealed a much higher binding constant for acetate than for chloride (see Table 1 and Figures S19 and S24).^59^ Unfortunately, addition of [Bu_4_N]^+^[AcO]^−^ to **SQ1** as well as [Bu_4_N]^+^[F]^−^ to both **SQ1** and **SQ2** resulted in severe peak broadening of the squaramide NH signals into the baseline (Figures S14, S15, and S20). Such an observation has previously been ascribed to either strong hydrogen bonding^47^ or fast proton exchange/deprotonation.^58^

**Table 1:** Binding Constants of Squaramide-Based Crosslinkers **SQ1** and **SQ2**^a^^,^^b^

	*K*_a_ [M^–1^]	
anion	**SQ1**	**SQ2**
I^–^	<10^c^	<10^c^
Br^–^	31.6	<10^c^
Cl^–^	354	67.5
AcO^–^	7.70 × 10^4^^d^	1.82 × 10^3^^e^
F^–^	1.96 × 10^5^^d^	1.03 × 10^3^^d^

a Tetrabutylammonium anions were added to **SQ1** and **SQ2** (5 mM) in DMSO/0.5% H_2_O.

b Errors are estimated to be no more than ±20%.

c Spectral changes were too minor to calculate a binding constant.

d Determined by UV–vis instead of ^1^H NMR titrations.

e Binding constant additionally determined by UV–vis titration as *K*_a_ = 1.11 × 10^3^ M^–1^.^59^

The stability constant of these monomer/anion combinations was therefore determined by UV–vis spectroscopic titrations in DMSO/0.5% H_2_O. When [Bu_4_N]^+^[AcO]^−^ was added to **SQ1**, the absorption maximum at λ = 365 nm decreased and bathochromically shifted to λ = 410 nm, in line with what has been previously reported for anion binding to squaramides.^60^ The titration data was successfully fitted to a 1:1 binding model using BindFit^61,62^ to afford an association constant that is nearly 2 orders of magnitude larger than the one determined for **SQ2** (see Table 1 and Figures S25, S28, S30, and S32).^59^ Addition of [Bu_4_N]^+^[F]^−^ to **SQ1** resulted in similar UV–vis spectral changes, while for **SQ2** mainly a decrease in absorption was observed (Figures S26 and S29). Fitting of the data to a 1:1 binding model gave affinities in the same range as determined for AcO^–^ binding to **SQ1** and **SQ2** (Table 1, Figures S31 and S33).^63^

To confirm that the observed spectral changes stem from binding rather than deprotonation,^64^**SQ1** was treated with excess [Bu_4_N]^+^[OH]^−^, revealing a much larger bathochromic shift (λ_max_ = 480 nm) than when [Bu_4_N]^+^[AcO]^−^ or [Bu_4_N]^+^[F]^−^ were added (Figure S27). In multiple examples reported in the literature, the absorption maximum of squaramide derivatives with aromatic substituents was found to red-shift to 460 nm,^47^ 485 nm,^65^ and 540 nm upon single deprotonation,^60,64^ while double deprotonation led to absorption above 600 nm.^64,65^ The much smaller bathochromic shifts observed upon addition of [Bu_4_N]^+^[AcO]^−^ or [Bu_4_N]^+^[F]^−^ to **SQ1** and **SQ2**, similar to what was reported earlier for anion binding to squaramide,^60^ thus suggest that neither of these crosslinkers experiences significant deprotonation during the UV–vis titration experiments.

Overall, the binding constants show an increase according to the anion series I^–^ < Br^–^ < Cl^–^ < AcO^–^ ∼ F^–^, covering 5 orders of magnitude. Most importantly, they correlate with the volume swelling ratios of our gels (*vide supra*). That is, samples immersed in [Bu_4_N]^+^[I]^−^, [Bu_4_N]^+^[Br]^−^, or [Bu_4_N]^+^[Cl]^−^ displayed concentration-independent swelling, reflecting the weak binding of the anions to **SQ1** and **SQ2**. On the other hand, [Bu_4_N]^+^[F]^−^ or [Bu_4_N]^+^[AcO]^−^ solutions containing anions with *K*_a_ > 1 × 10^3^ M^–1^ gave enhanced swelling behavior, resulting in the peak-shaped profiles shown in Figure 2.

### Reversible Swelling and Actuation

**Figure 3:**
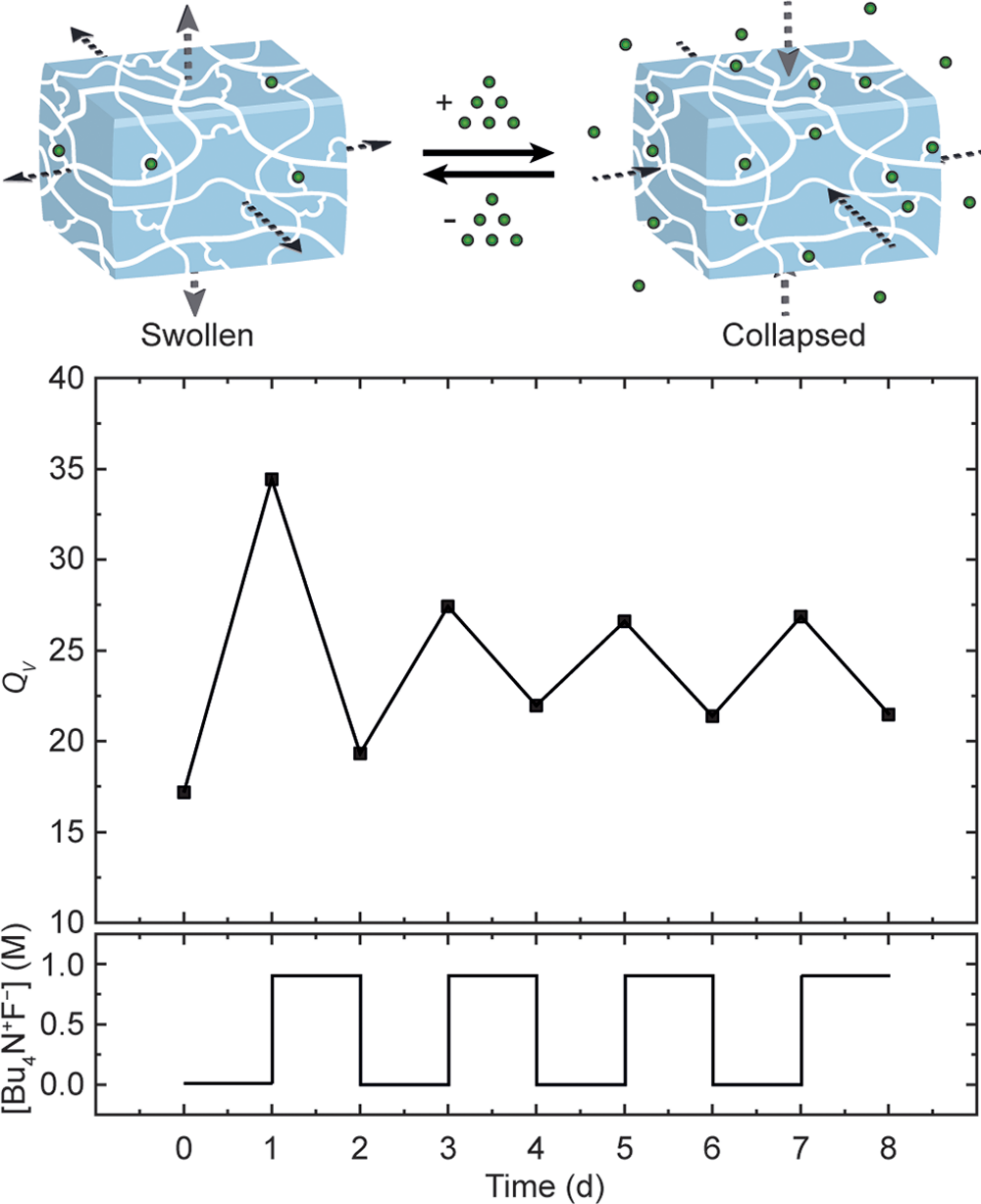
Volume swelling ratios of **SQ1**-crosslinked gel sample after synthesis (day 0), immersed in a 0.01 M [Bu_4_N]^+^[F]^−^ solution (day 1) and periodically swollen/collapsed in in 0.8 and 0 M [Bu_4_N]^+^[F]^−^ solutions, respectively (days 2–8).

To assess whether the anion-induced swelling process was reversible, the gel specimens were sequentially immersed in solutions with different salt concentrations. The polymeric networks crosslinked with **SQ1** were used in these experiments as they showed moderate stiffness and a large anion response. A gel sample that was first immersed in a 0.01 M solution of [Bu_4_N]^+^[F]^−^ for 24 h reached a volume swelling ratio of *Q*_*V*_ = 34.4. Subsequent immersion in a 0.8 M [Bu_4_N]^+^[F]^−^ solution induced shrinking to give a value of *Q*_*V*_ = 19.3, which is in agreement with what was observed at the higher concentrations in the gel swelling experiments (*vide supra*). Further alternation between 0.8 M [Bu_4_N]^+^[F]^−^ and salt free solutions led to repeated swelling and shrinking exhibiting *Q*_*V*_ values of approximately 21.5 and 26.5, respectively (Figure 3). These values correspond well with the concentration-dependent gel swelling experiments, which demonstrated minimum and maximum volume swelling ratios *Q*_*V*_ = 19.1 and *Q*_*V*_ = 26.1, respectively. This periodic swelling experiment was additionally carried out over longer immersion time (63 days with intervals of 7 days) with solutions of either [Bu_4_N]^+^[F]^−^ or [Bu_4_N]^+^[AcO]^−^, showing similar expansion and shrinking behavior (Figure S54).

**Figure 4:**
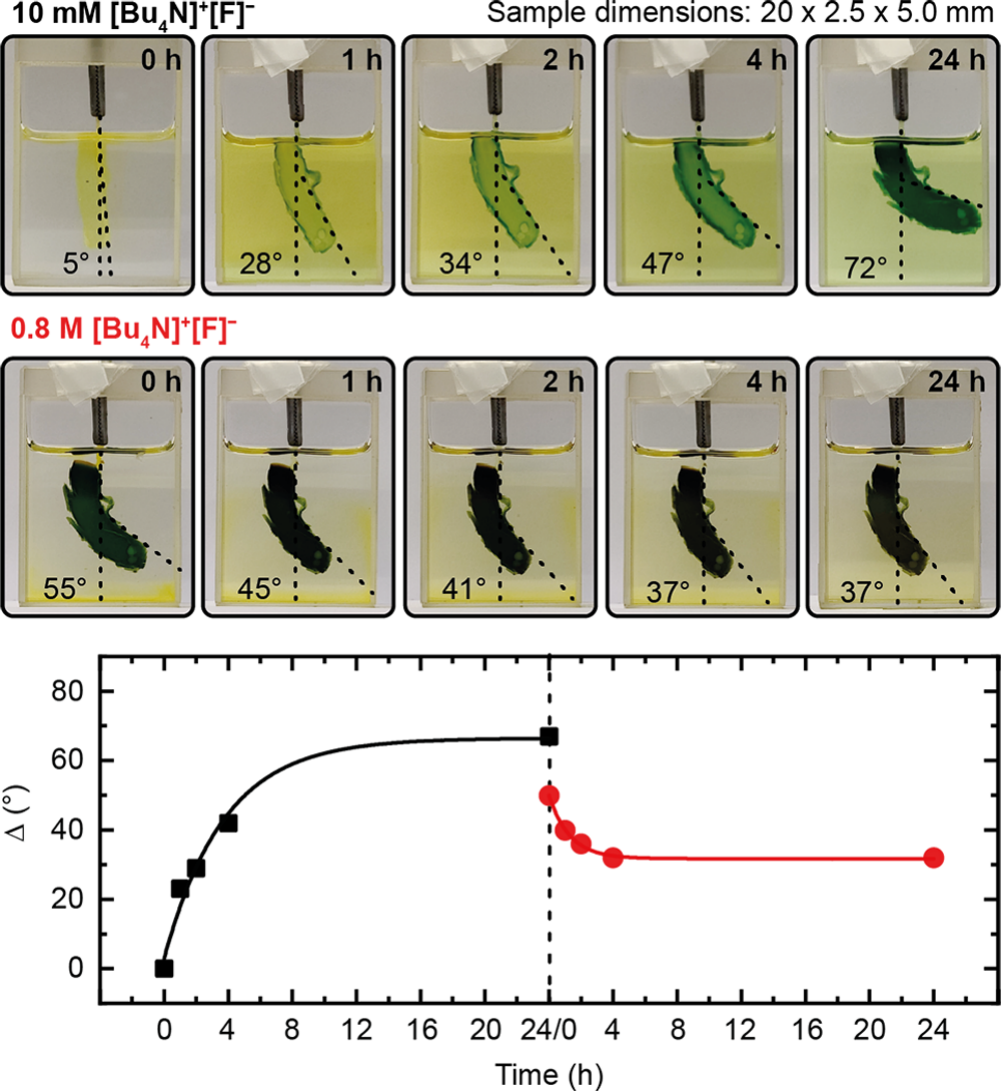
Actuation experiment using **SQ1**-crosslinked gel (*L* × *W* × *H* = 20 × 2.5 × 5.0 mm, 5.0 mol % crosslinker) showing pictures of the bending specimen over time and the degree of bending δ plotted vs time. Lines are drawn manually to guide the eye. Please note that, at this crosslinker concentration, the gel is prone to abrasion, which can be seen in the pictures.

Next, we became interested in studying whether nonuniform, anisotropic anion-induced swelling could result in bending and actuation of the gel specimen. To achieve this, a solid support was introduced into the polymeric network. A PEEK mold was used to fabricate rod-shaped gel samples (5 mol % crosslinker **SQ1**), with a thin strip of cellulose filter paper embedded into the gel (Figure S55). Once the gel sample was fully cured, it was removed from the mold and cut into dimensions of 20 × 2.5 × 5.0 mm (*L* × *H* × *W*). Then, the gel-embedded filter strip was fixed by using a clamped pair of tweezers, and the gel sample was immersed in a [Bu_4_N]^+^[F]^−^ solution (Figure S56). Initially, at a concentration of 0.01 mM, the sample was nearly straightened (5°), yet as time progressed the bending angle increased to a maximum of 72° after 24 h (Figure 4).

The process of bending was accompanied by an intense green coloration of the gel similar to what was observed in the swelling experiments. When the original immersion solution was replaced by a highly concentrated one of 0.8 M [Bu_4_N]^+^[F]^−^, the bending angle retreated to 37° after 24 h. A plot of the degree of bending against time showed an asymptotic behavior in both instances (bending and straightening, Figure 4). Importantly, when a salt free solution was used at the start (instead of 0.01 M [Bu_4_N]^+^[F]^−^), the degree of bending reached a plateau already at 55° (Figure S57). Moreover, bending was less reversible upon replacement with the highly concentrated 0.8 M [Bu_4_N]^+^[F]^−^ solution, resulting in an angle of 39°. This observation is in line with the same volume swelling ratios determined for the salt free solution and the highly concentrated salt solutions.^66^

By reducing the crosslinker concentration to 2.5 mol %, a final bending angle of 82° was achieved after 24 h of immersion in the 0.01 M [Bu_4_N]^+^[F]^−^ solution, which was partially reversed by 12° after replacing it for the 0.8 M solution (Figure S58). In comparison, a control sample that was first immersed in a salt free solution (as opposed to 0.01 mM [Bu_4_N]^+^[F]^−^) did not show any reversibility (Figure S59). It should be noted that, despite the low quantities of anion-binding crosslinker (2.5–5.0 mol %) in these polymeric gels, the effect of bending was readily perceivable. Overall, the higher crosslinker concentration narrowed the bending angle boundaries (because of making the material stiffer), improving the reversibility of the anion-induced bending process.

## Conclusions

In summary, we have demonstrated the capacity of two different squaramide crosslinkers to infuse polymeric gel networks with anion-responsive properties. These networks were successfully prepared by using small amounts (2.5–5.0 mol %) of the crosslinker together with DMAAm as the main monomer in a free radical polymerization process. The volume swelling ratio of the hence obtained gels (determined after 24 h and 7 days) showed peak-shaped dependency on the concentration of the most strongly binding anions (that is, F^−^ and AcO^−^). In solutions of non- and weakly associating anions, the gels did not show such concentration-dependent swelling and behaved as if they were absent of the anion-binding motif. The observed swelling enhancement is a result of the networks’ ability to immobilize anions as was supported by binding studies, which revealed the highest association constants for the anions that displayed the largest volume swelling ratios. Partial reversibility of swelling was demonstrated by alternate immersion of gel specimens in respective high and low concentrated solutions of the stronger binding anions. By embedding a solid support into the gels to cause nonuniform swelling, chemically responsive soft actuators were created. That is, successive immersion of solid-supported rod-shaped gel specimens into low and high concentrated solutions of [Bu_4_N]^+^[F]^−^ gave rise to reversible bending, which was monitored over time. Additionally, the anion-induced swelling and bending process was accompanied by an intense green coloration of the gel, which may be applicable for sensing and detection purposes. Our results demonstrate that squaramide crosslinkers enable very effective anion-responsive swelling as well as actuation of soft polymer gels, which opens the path toward materials and robots that autonomously react with motion and a change in physical appearance to the anionic species that surround them.

## Experimental Section

### Materials

Unless otherwise indicated, all solvents were purchased from commercial sources and were used without further purification. 3,4-Diethoxycyclobut-3-ene-1,2-dione (95%, ABCR), zinc trifluoromethanesulfonate (98%, Sigma-Aldrich), 4-vinylaniline (97%, Sigma-Aldrich), 2-aminoethyl methacrylate hydrochloride (90%, Sigma-Aldrich), *N*,*N*-diisopropylethylamine (DIPEA, >99%, Sigma-Aldrich), *N*,*N*-dimethylacrylamide (DMAAm, 99%, Sigma-Aldrich), ethylene glycol dimethacrylate (EGDMA, 98%, Sigma-Aldrich), 2,2′-azobis(2-methylpropionitrile) (AIBN, 98%, Sigma-Aldrich), tetrabutylammonium fluoride hydrate ([Bu_4_N]^+^[F]^−^, 98%, ABCR), tetrabutylammonium chloride ([Bu_4_N]^+^[Cl]^−^, >97%, Sigma-Aldrich), tetrabutylammonium bromide ([Bu_4_N]^+^[Br]^−^, >98%, Sigma-Aldrich), tetrabutylammonium iodide ([Bu_4_N]^+^[I]^−^, 98%, Sigma-Aldrich), and tetrabutylammonium acetate ([Bu_4_N]^+^[AcO]^−^, 95%, ABCR) were used as received. AIBN was recrystallized from methanol prior to use and stored below 5 °C. To remove the residual inhibitor, the main monomer DMAAm was destabilized via a short column of basic alumina prior use.

### Instrumentation

^1^H and ^13^C NMR spectra were recorded on a Bruker Avance 500 NMR spectrometer (500 and 126 MHz, respectively) and are reported as follows: chemical shift δ (ppm) (multiplicity, coupling constant *J* (Hz), number of protons, assignment). The residual protiated solvent signals (DMSO, δ_H_ = 2.50 ppm, δ_C_ = 39.5 ppm) were used as reference. Chemical shifts are reported in ppm to the nearest 0.01 ppm for ^1^H and the nearest 0.1 ppm for ^13^C. Infrared spectra were recorded on a PerkinElmer FT-IR Spectrum Two spectrometer using an ATR unit. Absorbance maxima are reported in wavenumbers (cm^–1^), and only selected intensities are reported. High-resolution mass spectrometry (HRMS) was performed on a Thermo Scientific Q Exactive HF spectrometer with electron spray ionization (ESI). Rheological data were recorded at a Discovery HR-2 hybrid rheometer (TA Instruments). For all measurements a sand-blasted plate stainless steel 8 mm SMART-SWAP plate (TA Instruments) was applied at a gap size between 700 and 1100 μm at 298 K. Samples were sliced into thin pieces using a razor blade and quickly loaded onto the rheometer as they slowly experienced syneresis over time. Oscillatory frequency sweeps were recorded at 1% strain in a frequency range between 0.1 and 100 Hz. Results were analyzed using TA Instruments TRIOS software. UV–vis spectra were recorded on an Agilent Cary 8454 spectrometer in a 1 cm quartz cuvette. High-resolution mass spectrometry (ESI-MS) was performed on a Thermo Scientific Q Exactive HF spectrometer with ESI ionization.

### Synthesis of **SQ1**

The procedure was adapted from the one reported by Manesiotis et al.^47^ Vinylaniline (0.72 mL, 6.17 mmol) was added to a stirred solution of 3,4-diethoxycyclobut-3-ene-1,2-dione (0.44 mL, 2.94 mmol) and zinc trifluoromethanesulfonate (213 mg, 0.588 mmol) in ethanol (11.8 mL, 0.25 M) at rt. The reaction mixture was stirred at rt for 24 h. Next, the light brown precipitate was filtered off, washed with a small amount of cold ethanol, and dried *in vacuo* to give product **SQ1** as a light brown powder (909 mg, 97%). Mp > 400 °C (decomp.).^1^H NMR (500 MHz, DMSO-*d*_6_) δ 9.93 (s, 2H; N*H*), 7.55–7.39 (m, 8H; C*H*_arom_), 6.70 (dd, *J* = 17.6, 10.9 Hz, 2H; C*H*), 5.77 (d, *J* = 17.6 Hz, 2H; *trans*–C*H*_2_), 5.20 (d, *J* = 10.9 Hz, 2H; *cis*-C*H*_2_). ^13^C NMR (126 MHz, DMSO-*d*_6_) δ 182.2 (*C*=O), 166.0 (=*C*NH), 138.7 (NH*C*), 136.5 (H*C*=CH_2_), 132.9 (C7), 127.8 (*p*-*C*_arom_), 119.1 (*o*-*C*_arom_), 113.7 (HC=*C*H_2_). IR (ATR): ν 3145 (m), 3092 (m), 3065 (m), 3007 (m), 1800 (m), 1795 (w), 1712 (s), 1676 (s), 1598 (s sh), 1561 (s), 1539 (s sh), 1518 (s sh), 1461 (s), 1439 (s sh), 1424 (w sh), 1409 (s), 1355 (m), 1328 (m), 1234 (m), 1188 (m), 1163 (w), 1130 (m), 1099 (w), 1080 (w), 1036 (w), 1017 (w), 995 (m), 968 (w), 907 (s), 863 (w), 838 (s), 823 (w sh), 809 (w), 784 (m), 774 (m), 752 (s), 726 (s), 661 (w), 640 (w), 622 (w), 592 (m), 559 (w), 491 (s). HRMS (ESI) *m*/*z*: 317.1284 (M + H)^+^, calculated for C_20_H_17_N_2_O2^+^: 317.1285.

### Synthesis of **SQ2**

DIPEA (2.15 mL, 12.3 mmol) was added dropwise over 1 h to a stirred suspension of 3,4-diethoxycyclobut-3-ene-1,2-dione (0.87 mL, 5.88 mmol) and 2-aminoethyl methacrylate hydrochloride (2.00 g, 12.0 mmol) in ethanol (24 mL, 0.25 M) at 0 °C. The reaction mixture was allowed to warm to rt and stirred for 24 h. Next, the reaction mixture was filtered, and the filtrate was concentrated under reduced pressure, redissolved in CH_2_Cl_2_ (50 mL), and washed with 1 M HCl_(aq)_ (2 × 50 mL). The organic layer was evaporated under reduced pressure, and the residual crude solid was redissolved in CH_2_Cl_2_ (10 mL). The resulting white suspension was filtered off to give product **SQ2** as a white powder (962 mg, 48%). Mp 163.1–166.2 °C. ^1^H NMR (500 MHz, DMSO-*d*_6_) δ 7.50 (br. s, 2H; N*H*), 6.01 (s, 2H; =C*H*_2_), 5.67 (s, 2H; =C*H*_2_), 4.20 (t, *J* = 5.1 Hz, 4H; OC*H*_2_), 3.87–3.69 (m, 4H; NHC*H*_2_), 1.85 (s, 6H; C*H*_3_). ^13^C NMR (126 MHz, DMSO-*d*_6_) δ 182.7 (*C*=O), 166.4 (O*C*=O), 135.7 (*C*=CH_2_), 126.0 (C=*C*H_2_), 64.2 (NH*C*H_2_), 42.3 (O*C*H_2_), 17.9 (*C*H_3_). IR (ATR): ν = 3174 (m br), 3089 (m br), 3047 (m br), 2971 (m br), 1807 (w), 1724 (s), 1641 (m), 1575 (s), 1489 (m), 1455 (m), 1442 (m), 1398 (m sh), 1362 (m), 1323 (w), 1297 (m), 1276 (m), 1202 (w sh), 1159 (s), 1036 (m), 1018 (m), 941 (m), 917 (w), 866 (w), 832 (m), 815 (m), 777 (br), 723 (m), 657 (w), 634 (w), 610 (w) cm^–1^. HRMS (ESI) *m*/*z*: 337.1391 (M + H)^+^, calculated for C_16_H_21_N_2_O_6_^+^: 337.1394; 359.1213 (M + Na)^+^, calculated for C_16_H_20_N_2_NaO_6_^+^: 359.1214.

### Exemplary Procedure for Polymer Gel Synthesis

DMAAm (1.04 mL, 10.1 mmol) was added to solution of **SQ1** (160 mg, 0.504 mmol, 5.0 mol %) and AIBN (16.6 mg, 0.101 mmol, 1.0 mol %) in DMSO (10.1 mL, [DMAAm] = 1 M) at room temperature. A volume of 250 μL of this gel precursor solution was then dispensed into 1 mL glass vials (40×) capped with a rubber seal. The solutions in these vials were degassed by purging with argon for 10 min. Finally, these solutions were heated to 60 °C overnight in an oven to give the cured anion-responsive networks as transparent slightly yellowish gels. Reactions with **SQ2** or ethylene glycol dimethacrylate (EGDMA) as crosslinkers were carried out analogously. For the gels that were used in actuation experiments, the gel precursor solution contained either 2.5 mol % or 5 mol % crosslinker.

## Supporting Information Available

^1^H and ^13^C NMR spectra of the title compounds, ^1^H NMR titrations, UV–vis titrations, gel characterizations, supporting swelling experiments, gravimetric statistics on gel samples, rheological data, additional cycling swelling experiments, and actuation experiments (PDF)

## Abbreviations

PMMA: poly(methyl methacrylate)
DMAAm: *N*,*N*-dimethylacrylamide
EGDMA: ethylene glycol dimethacrylate
