# Supplementary material for: Anion-Induced Reversible Actuation of Squaramide-Crosslinked Polymer Gels
Source: ACS Appl Mater Interfaces. 2022 Sep 13;14(38):43711–8. doi: 10.1021/acsami.2c11136 (PMC9523616; doi:10.1021/acsami.2c11136)
Supplement: Supplementary file 1 — am2c11136_si_001.pdf [file am2c11136_si_001.pdf]

SUPPORTING INFORMATION

Anion-Induced Reversible Actuation of Squaramide-Crosslinked Polymer Gels

Stefan Mommer, and Sander J. Wezenberg*

Leiden Institute of Chemistry, Leiden University,
Einsteinweg 55, 2333 CC Leiden, The Netherlands

Email: s.j.wezenberg@lic.leidenuniv.nl

Table of Contents

1 ^{1}H and ^{13}C Spectra of Title Compounds S2

2 Spectroscopic Titrations S7

3 Gel Characterization S23

4 Cycling Swelling Experiments S34

5 Rod-like Gels using a PEEK Mold S35

6 Actuation Experiments S36

7 References S39

1 ^{1}H and ^{13}C Spectra of Title Compounds

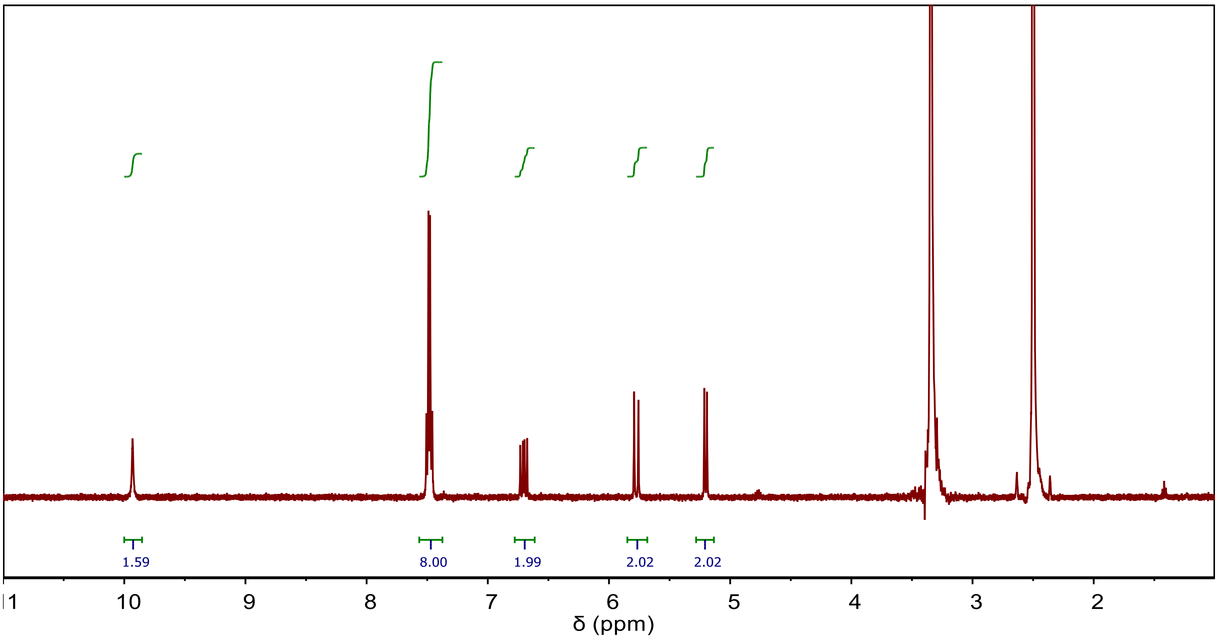

Figure S1. 500 MHz ^{1}H NMR spectrum of **SQ1** measured at 298 K in DMSO-d_6. Residual solventsignals: DMSO (*), H_2O (#).

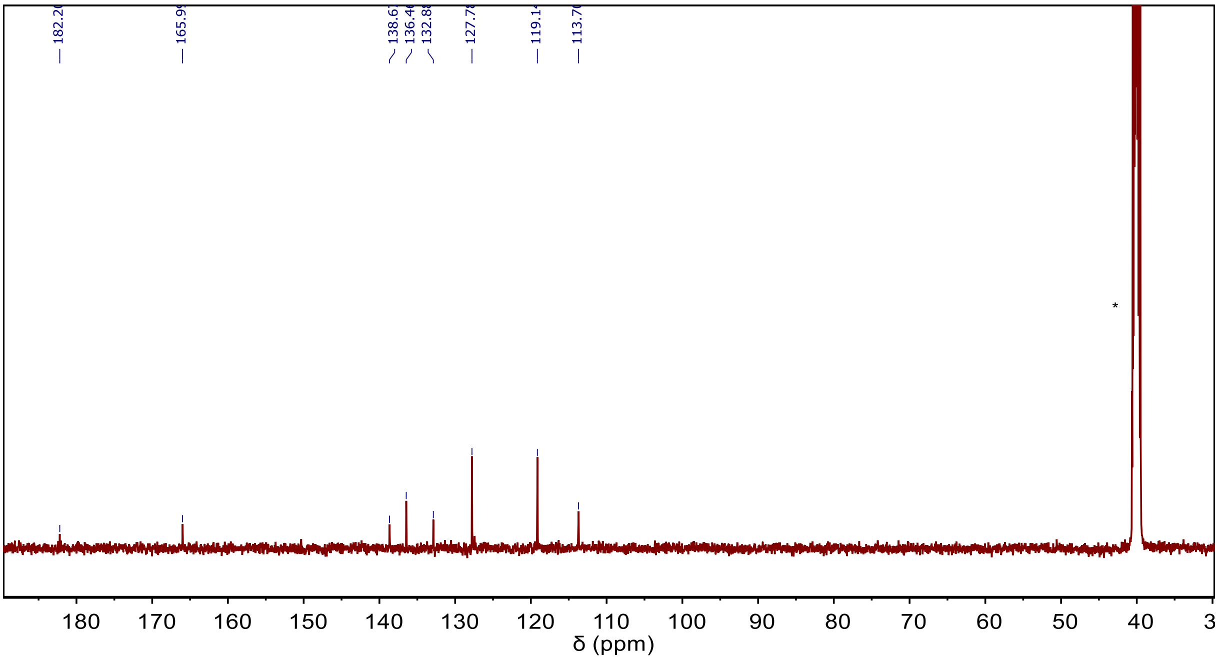

Figure S2. ^{13}C NMR spectrum of **SQ1** measured at 298 K in DMSO-d_6. Residual solvent signals: DMSO (*).

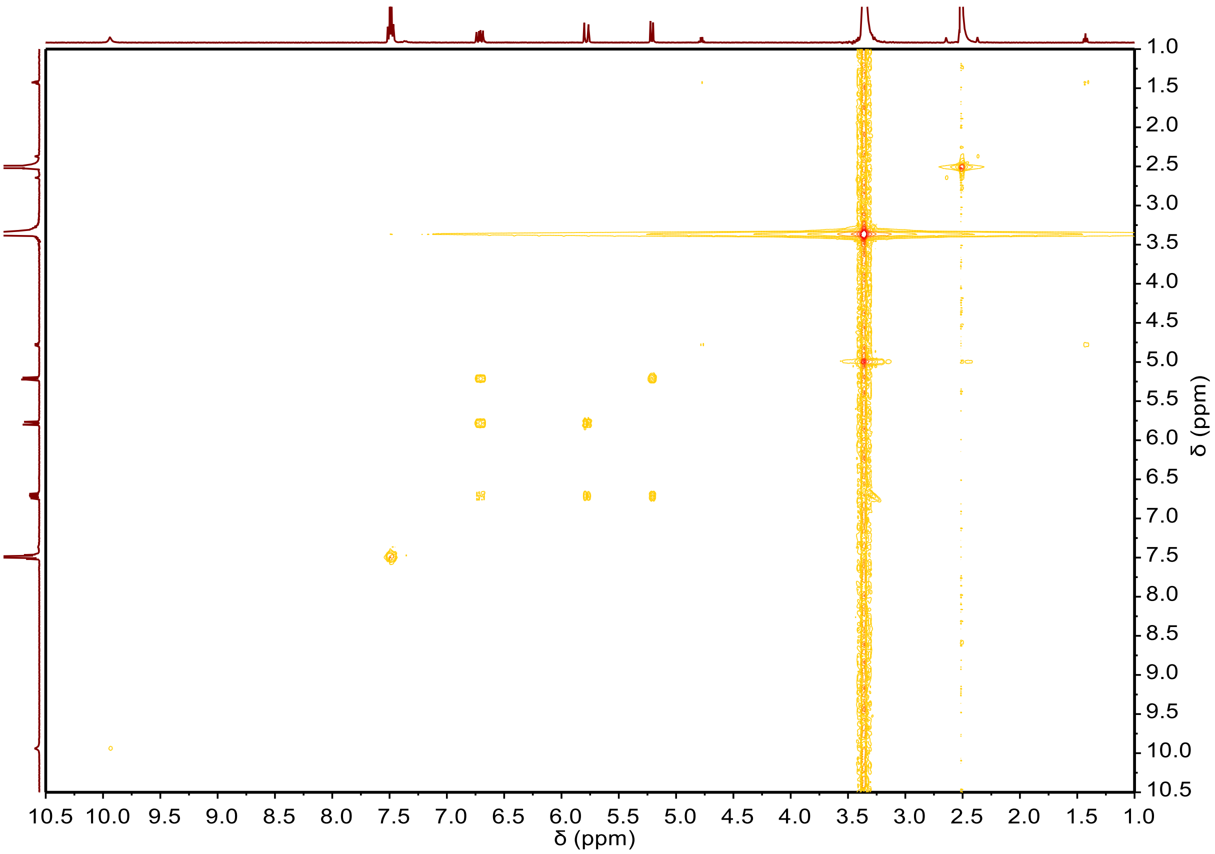

Figure S3. $^{1}H,^{1}H$ COSY spectrum of **SQ1** measured at 298 K in DMSO-d_6.

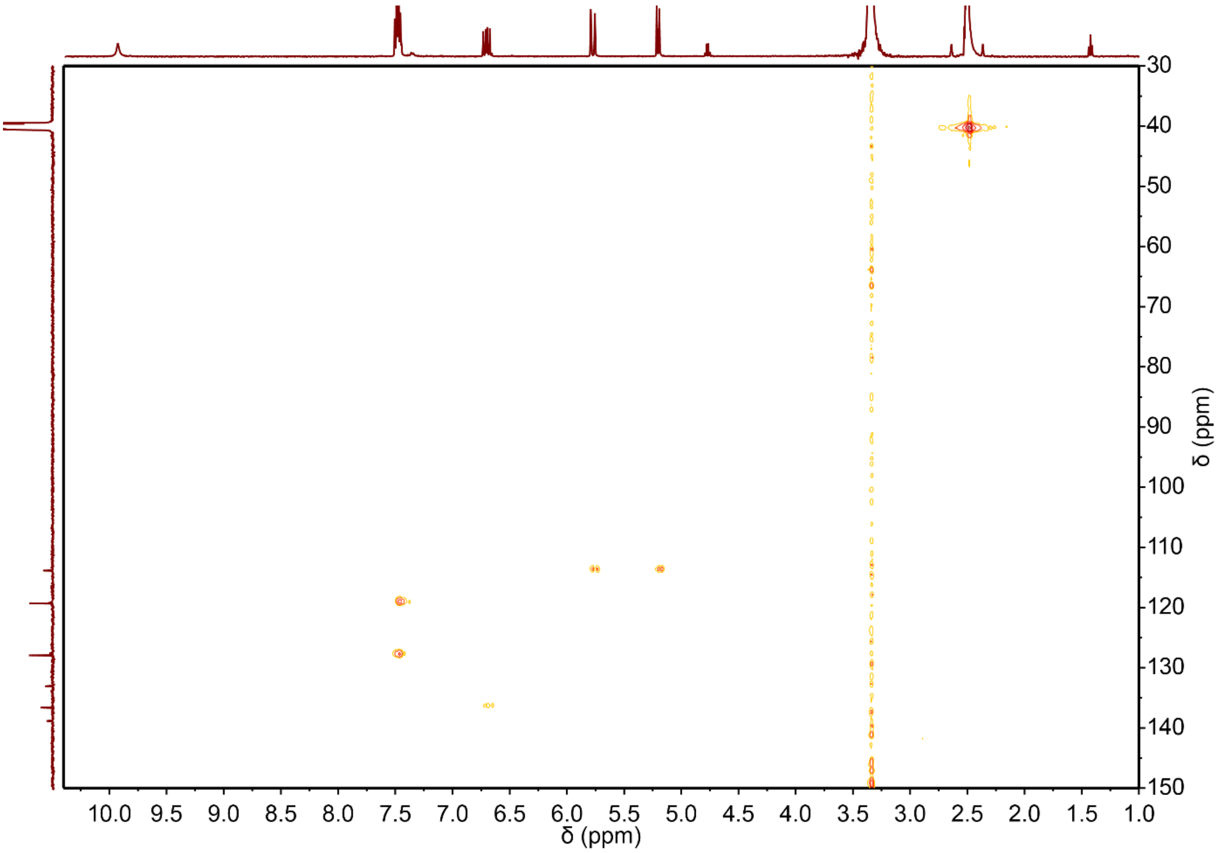

Figure S4. $^{1}H,^{13}C$ HSQC spectrum of **SQ1** measured at 298 K in DMSO-d_6.

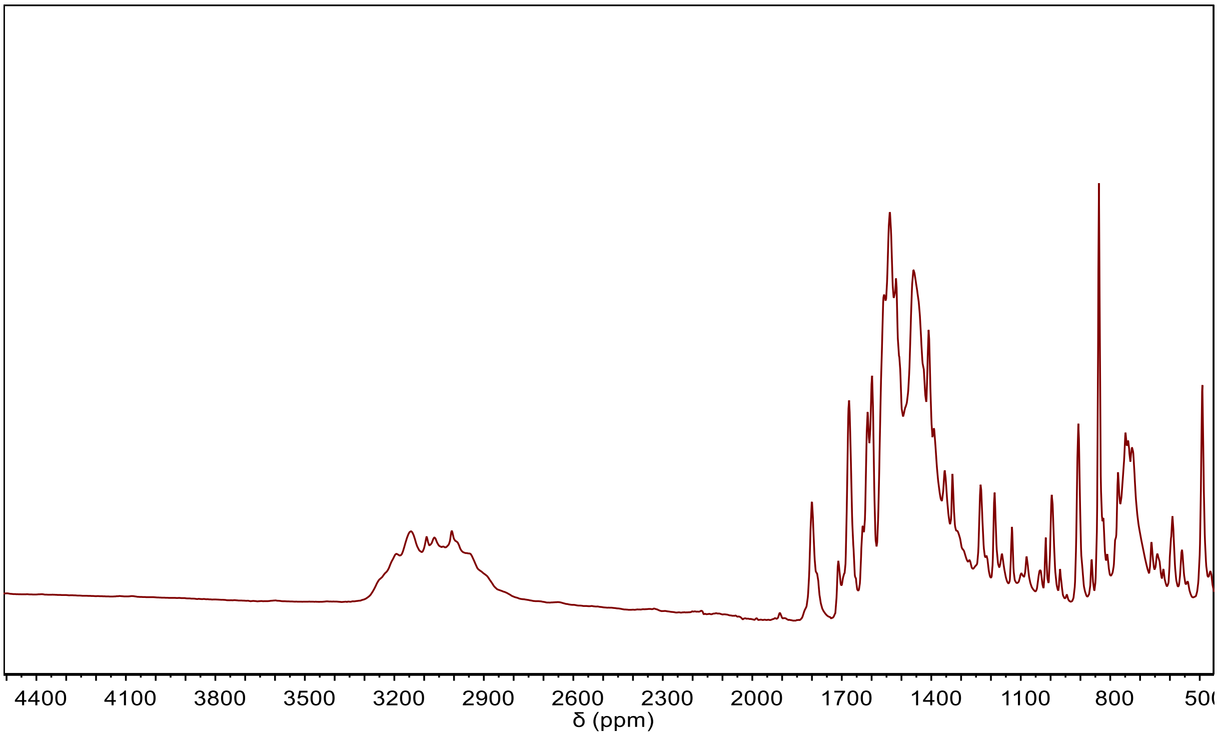

Figure S5. FT-IR spectrum of **SQ1**.

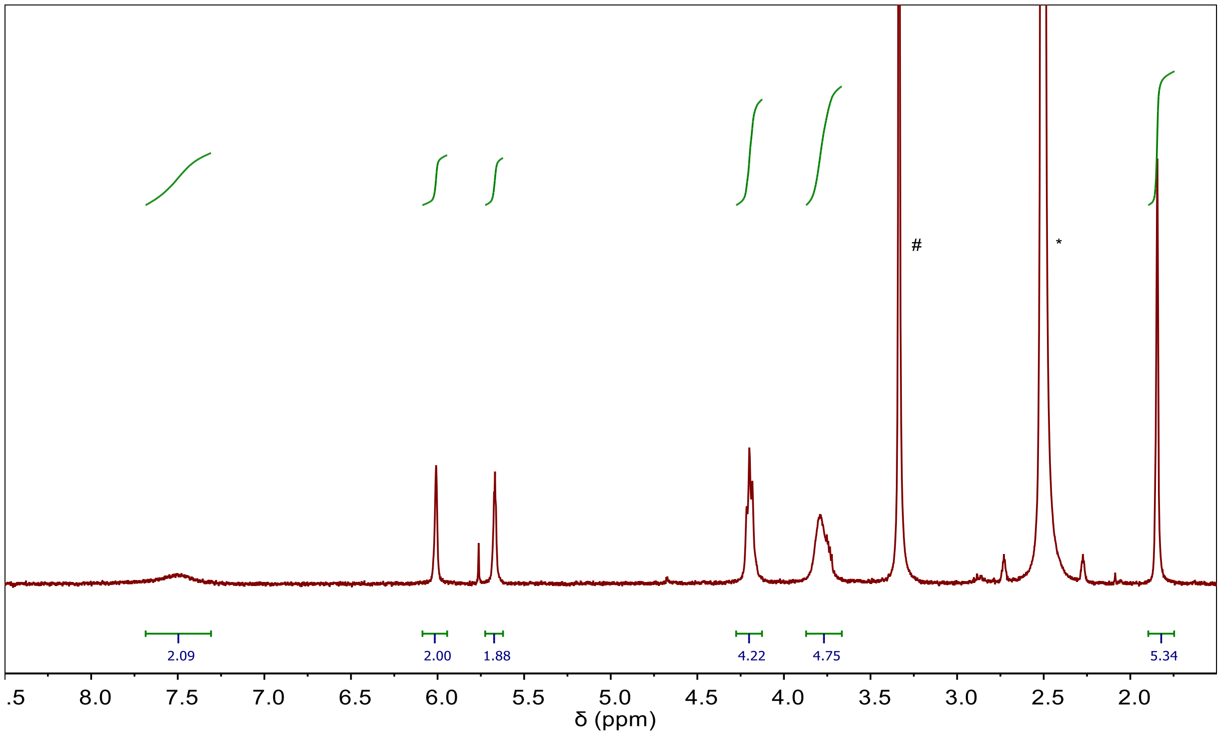

Figure S6. 500 MHz ^{1}H NMR spectrum of **SQ2** measured at 298 K in DMSO-d_6. Residual solvent peaks: DMSO (*), H_2O (#).

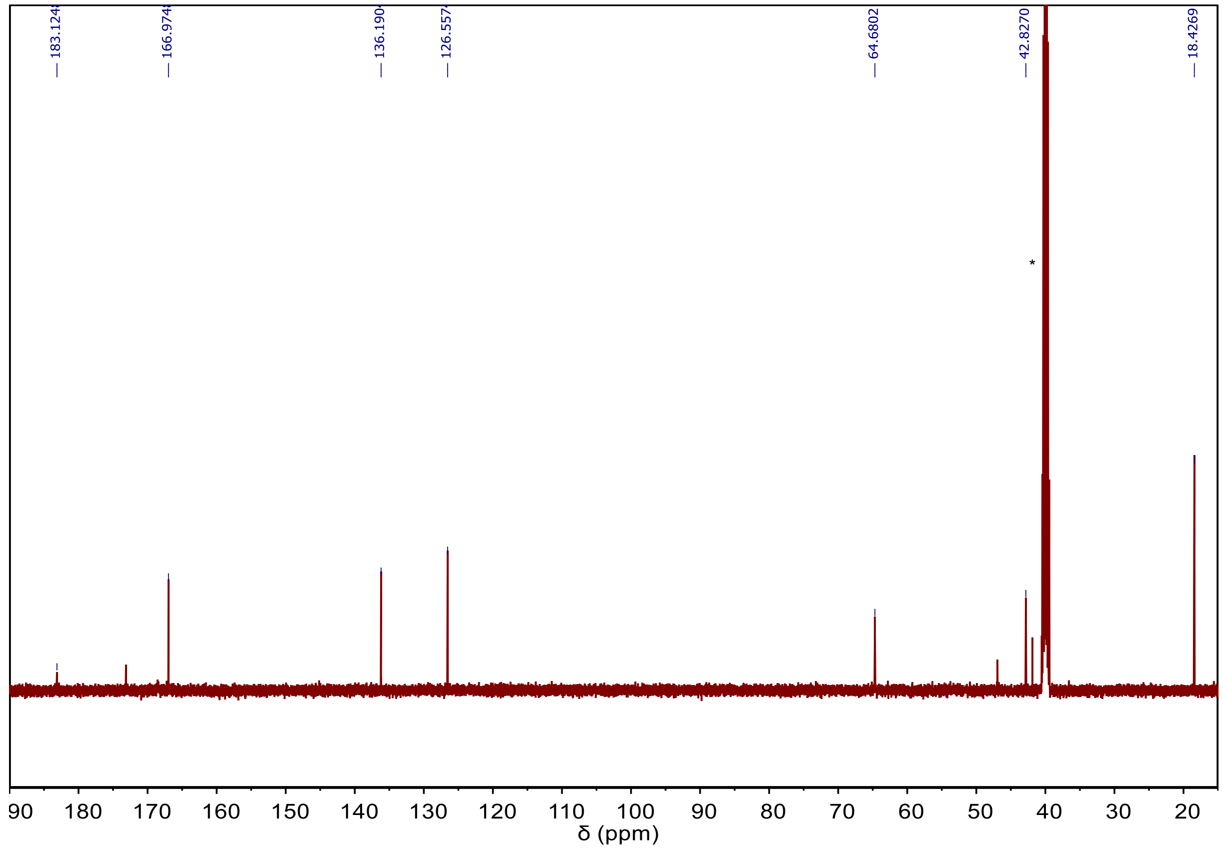

Figure S7. ^{13}C NMR spectrum of **SQ2** measured at 298 K in DMSO-d_6. Residual solvent signals: DMSO (*).

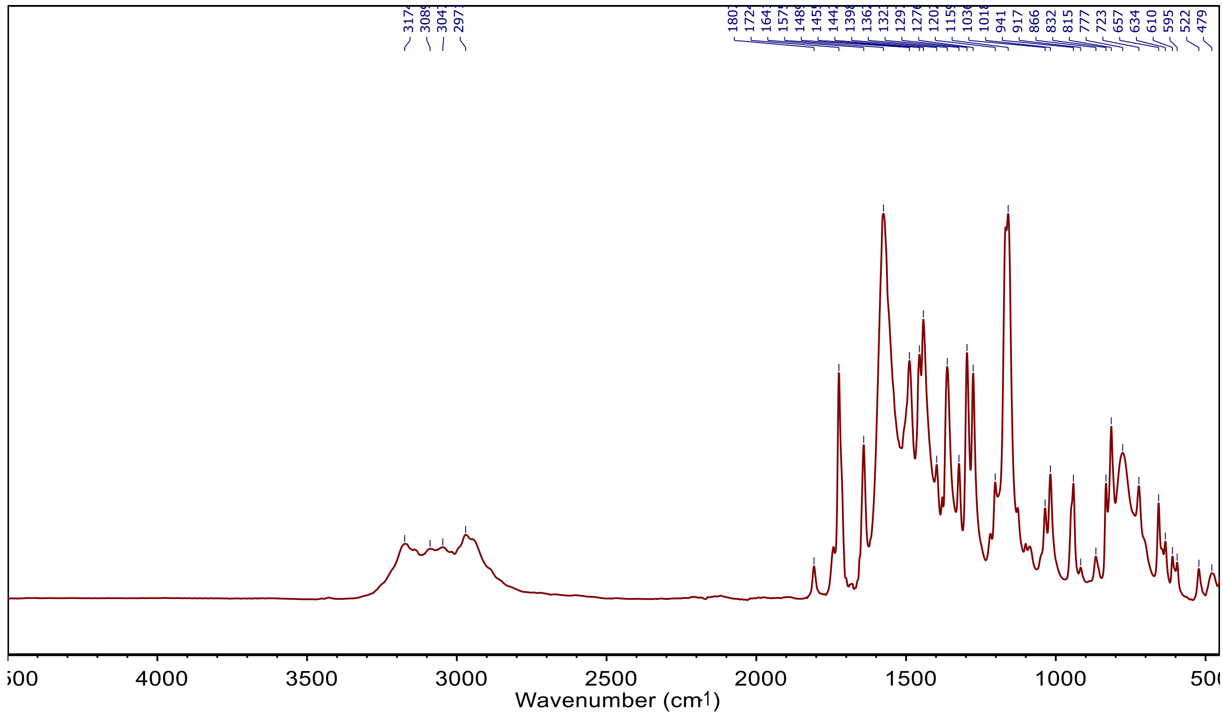

Figure S8. FT-IR spectrum of **SQ2**.

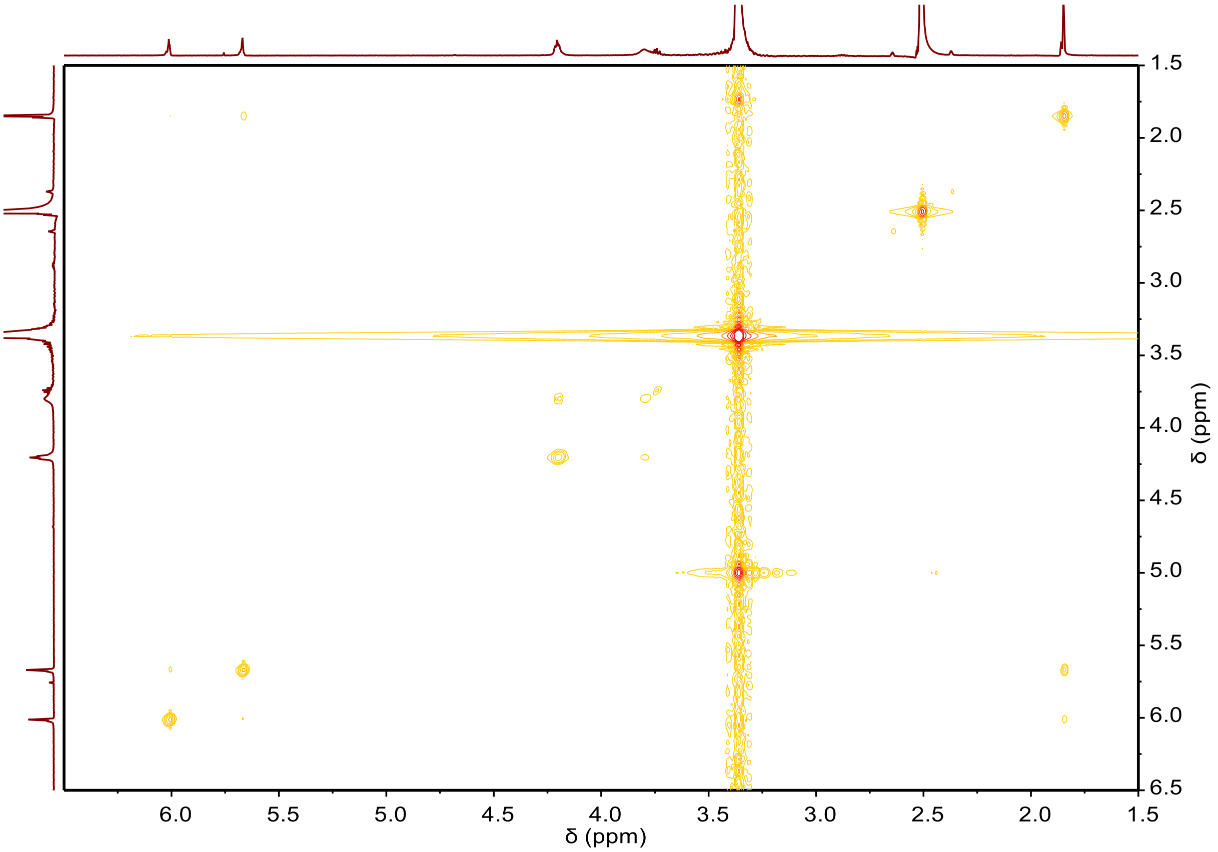

Figure S9. ^{1}H,^{1}H COSY spectrum of **SQ2** measured at 298 K in DMSO-d_6.

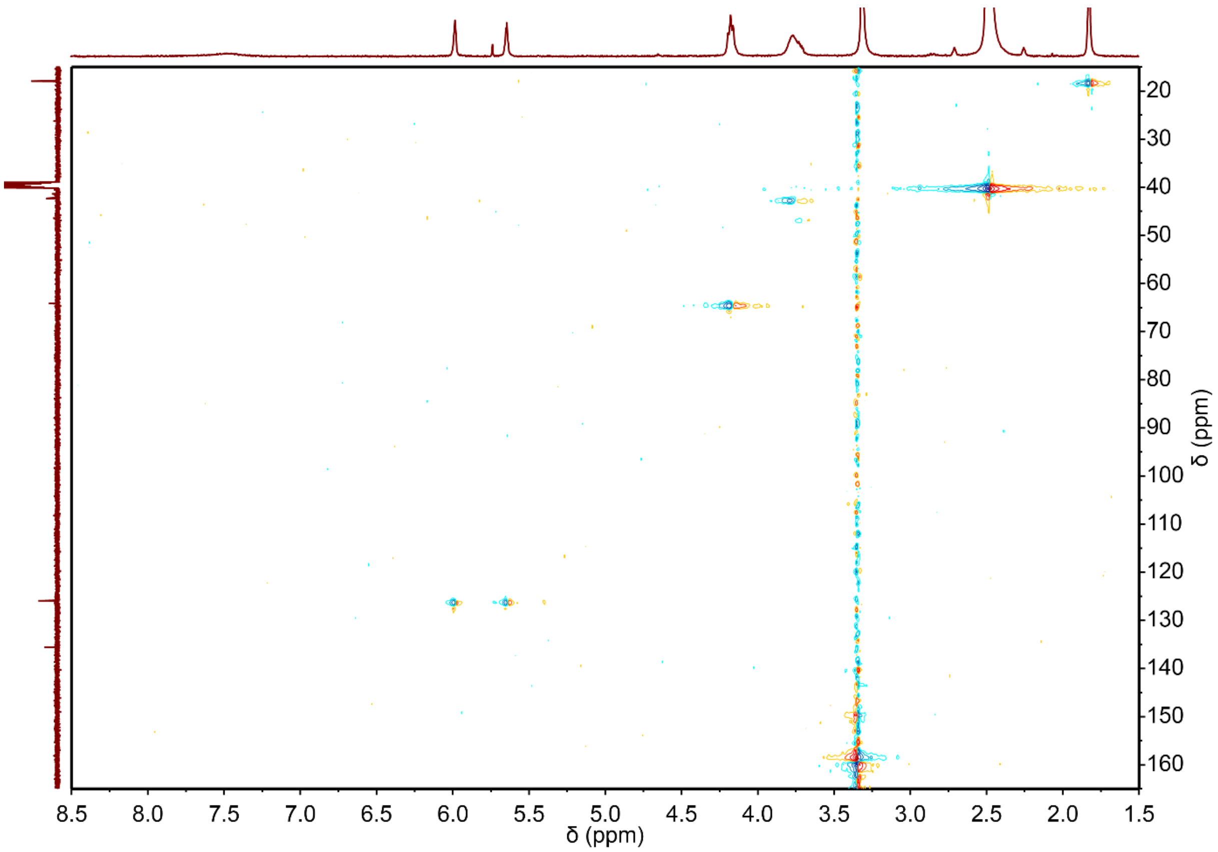

Figure S10. ^{1}H,^{13}C HSQC spectrum of **SQ2** measured at 298 K in DMSO-d_6.

2 Spectroscopic Titrations

2.1 ^{1}H NMR Titrations

For ^{1}H NMR titrations, 5 mM solutions of receptors (**SQ1** and **SQ2)** and 50 mM solutions of the tetrabutylammonium anion were prepared in a mixture of DMSO-d_6 and 0.5% H_2O (v/v). Subsequently, the 50 mM anion solution was added stepwise to 0.5 mL of the 5 mM receptor solution and a ^{1}H NMR spectrum (500 MHz, 298 K) was recorded after each addition. The addition steps constituted volumes of 10 x 10 mL, 5 x 20 mL, 2 x 50 mL and 2 x 100 mL adding up to a total volume of 500 mL guest solution added. Data was fitted to a 1:1 binding model using HypNMR 2008 (Version 4.0.68).[1]

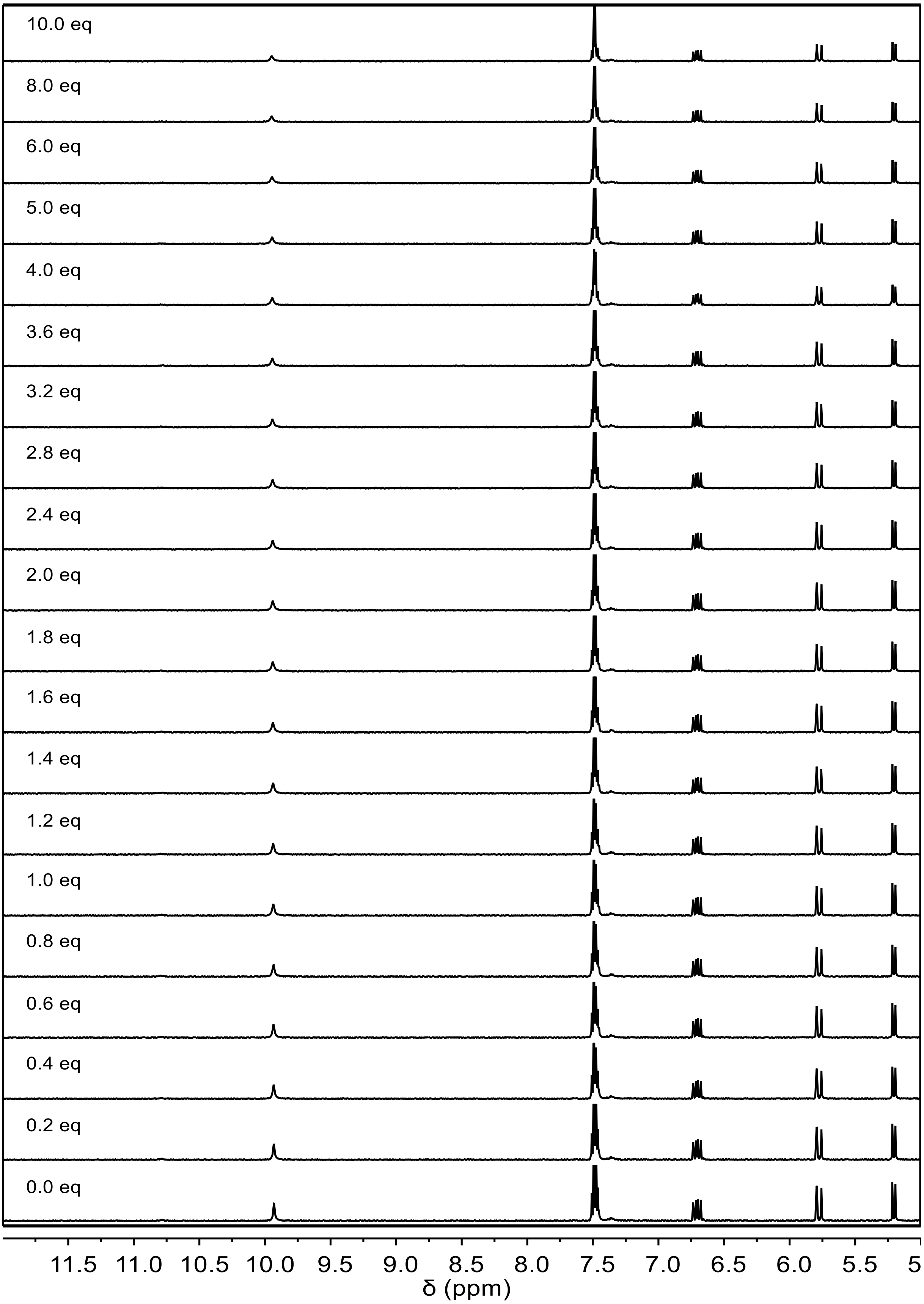

Figure S11. ^{1}H NMR spectral changes in the aromatic region of **SQ1** (DMSO-d_6/0.5%H_2O) upon the stepwise addition of $[Bu_4N]^+[I]^-$.

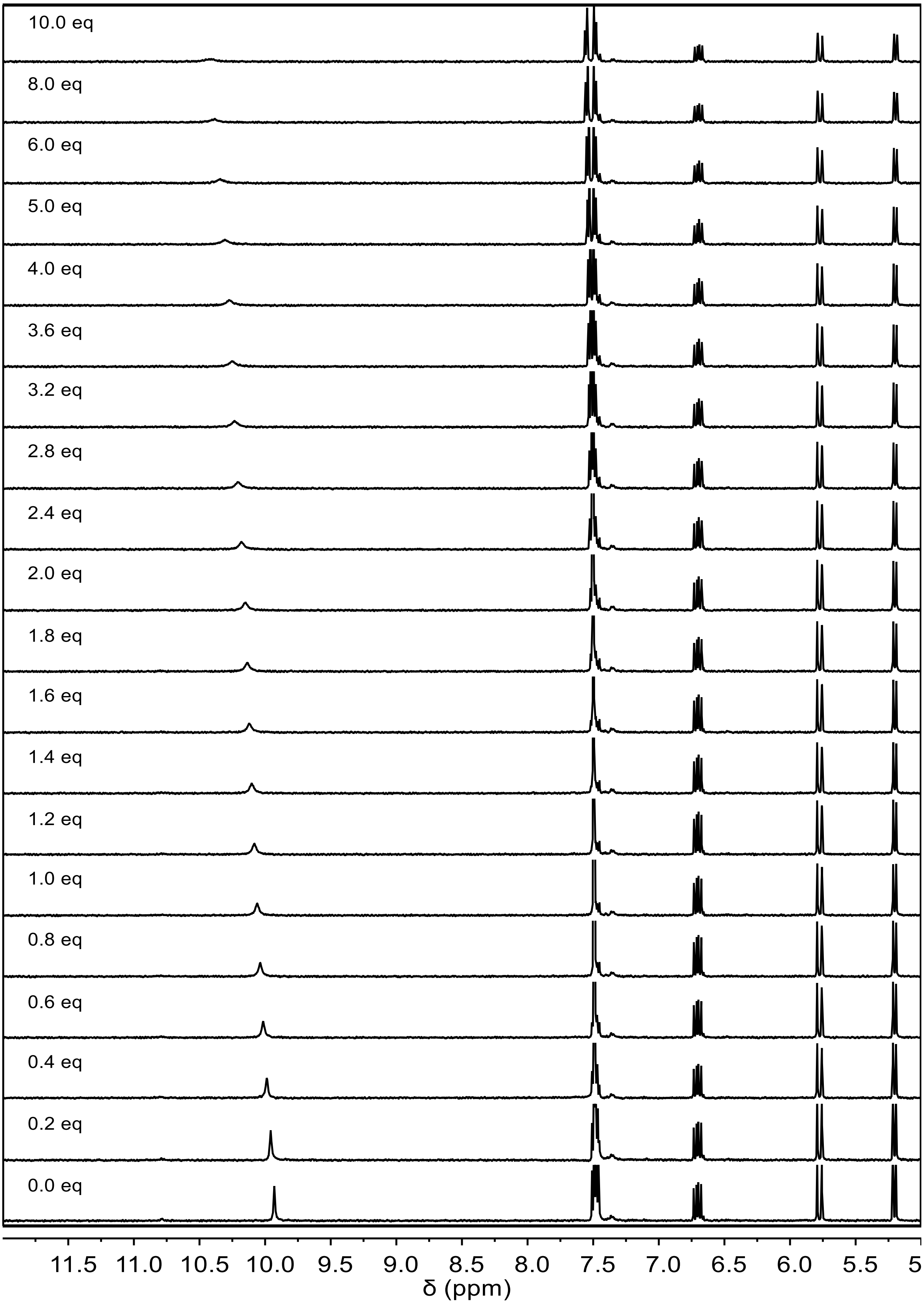

Figure S12. ^{1}H NMR spectral changes in the aromatic region of **SQ1** (DMSO-d_6/0.5%H_2O) upon the stepwise addition of $[Bu_4N]^+[Br]^-$.

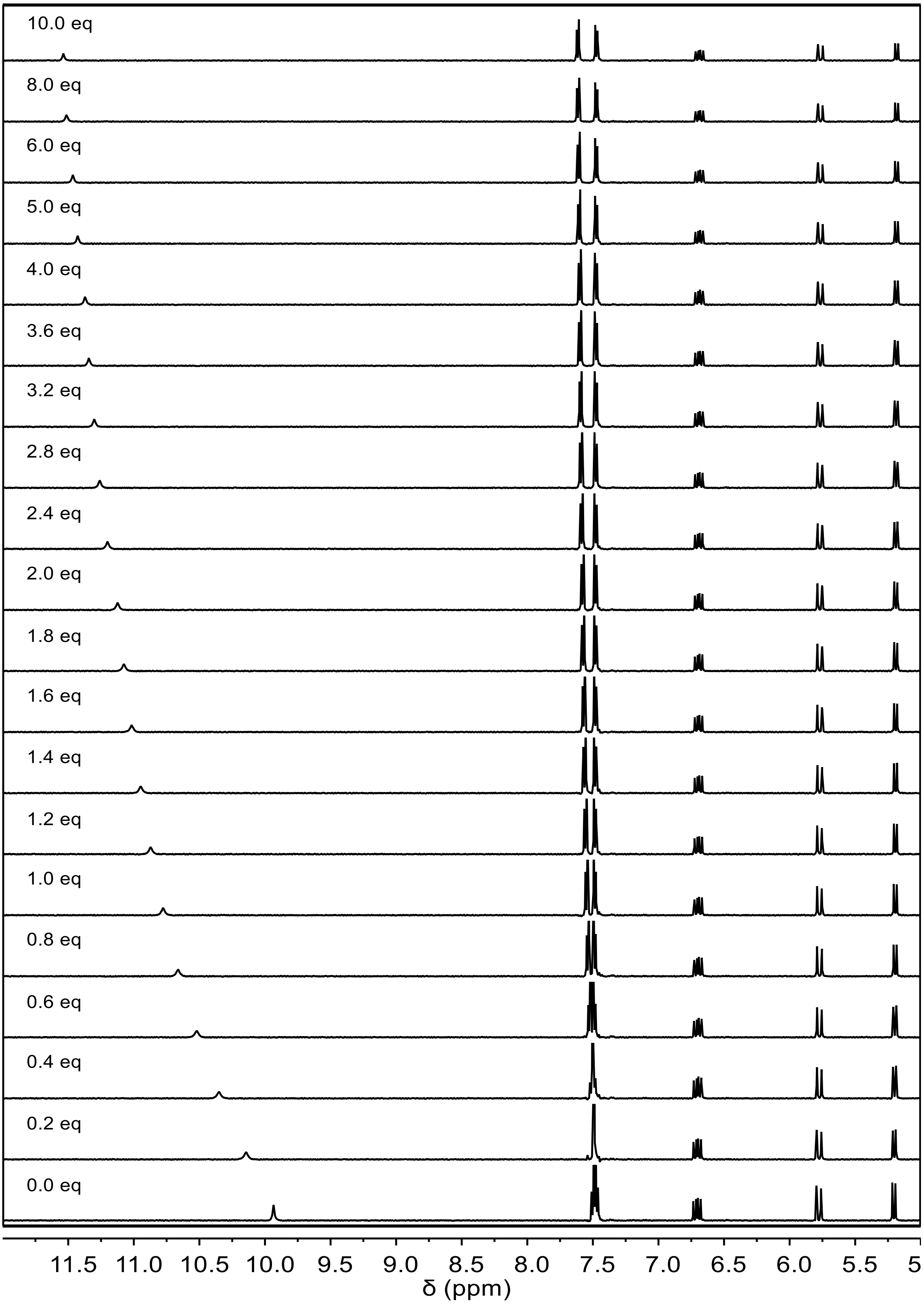

Figure S13. ^{1}H NMR spectral changes in the aromatic region of **SQ1** (DMSO-d_6/0.5%H_2O) upon the stepwise addition of $[Bu_4N]^+[Cl]^-$.

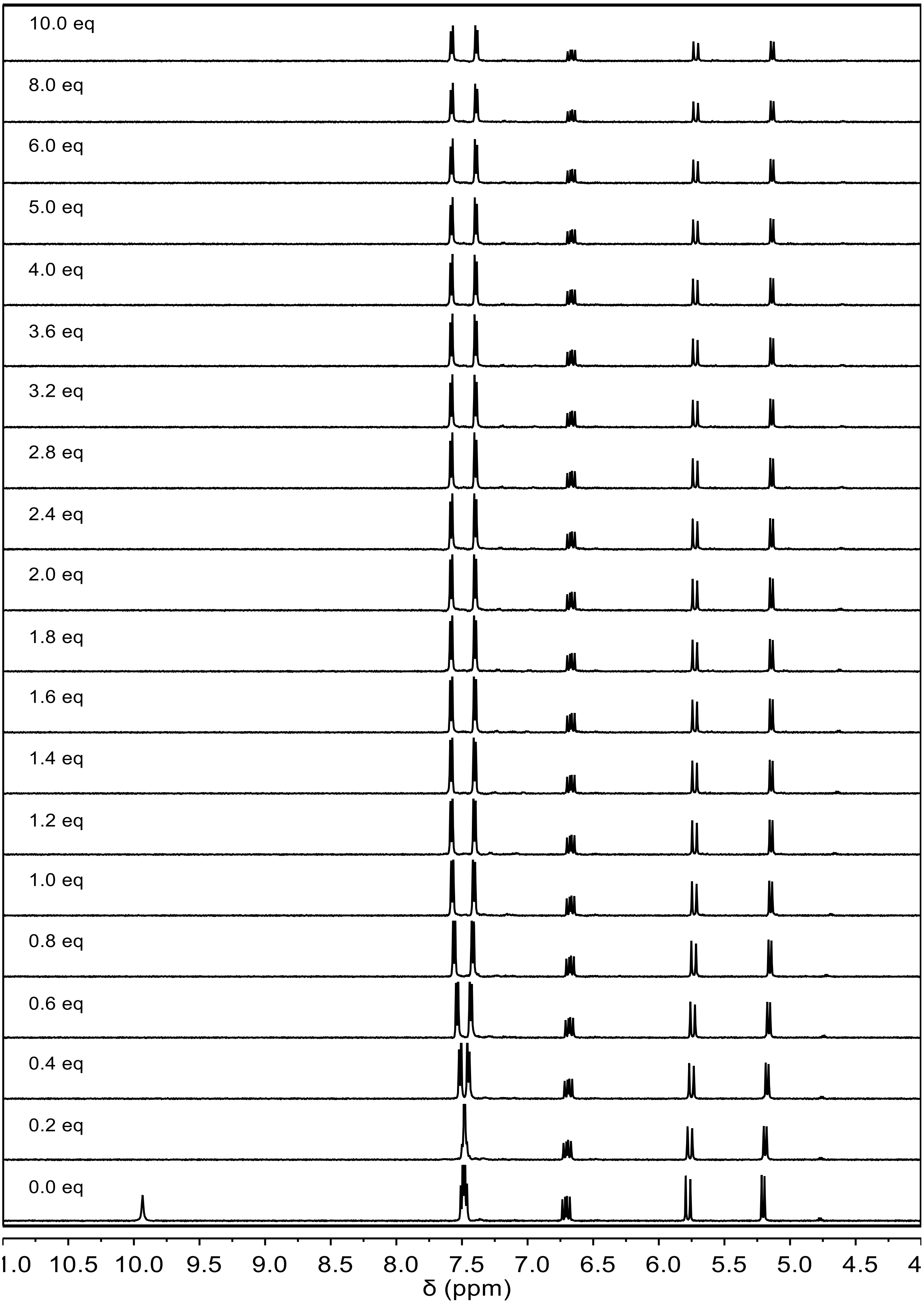

Figure S14. ^{1}H NMR spectral changes in the aromatic region of **SQ1** (DMSO-d_6/0.5%H_2O) upon the stepwise addition of $[Bu_4N]^+[AcO]^-$.

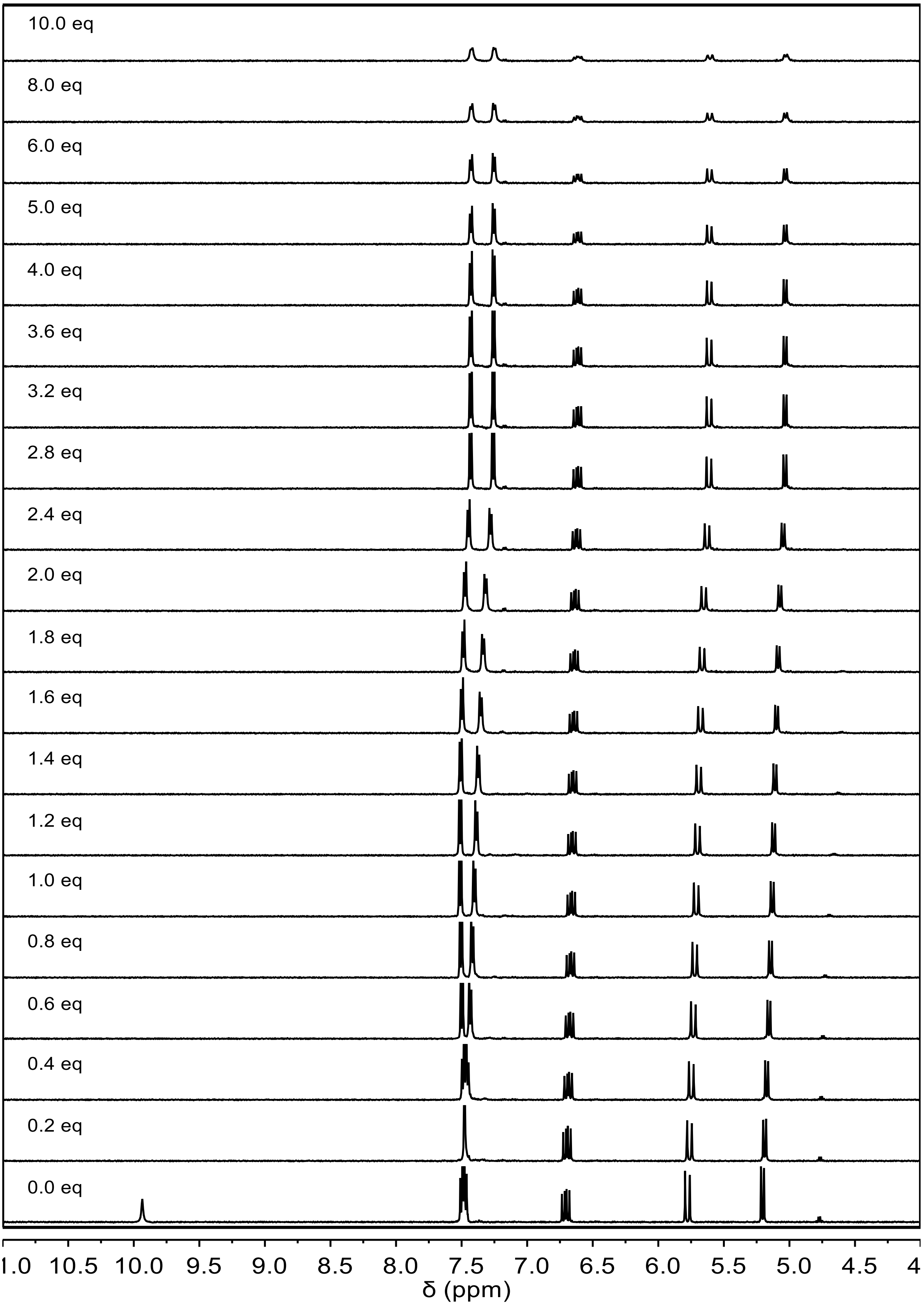

Figure S15. ^{1}H NMR spectral changes in the aromatic region of **SQ1** (DMSO-d_6/0.5%H_2O) upon the stepwise addition of $[Bu_4N]^+[F]^-$.

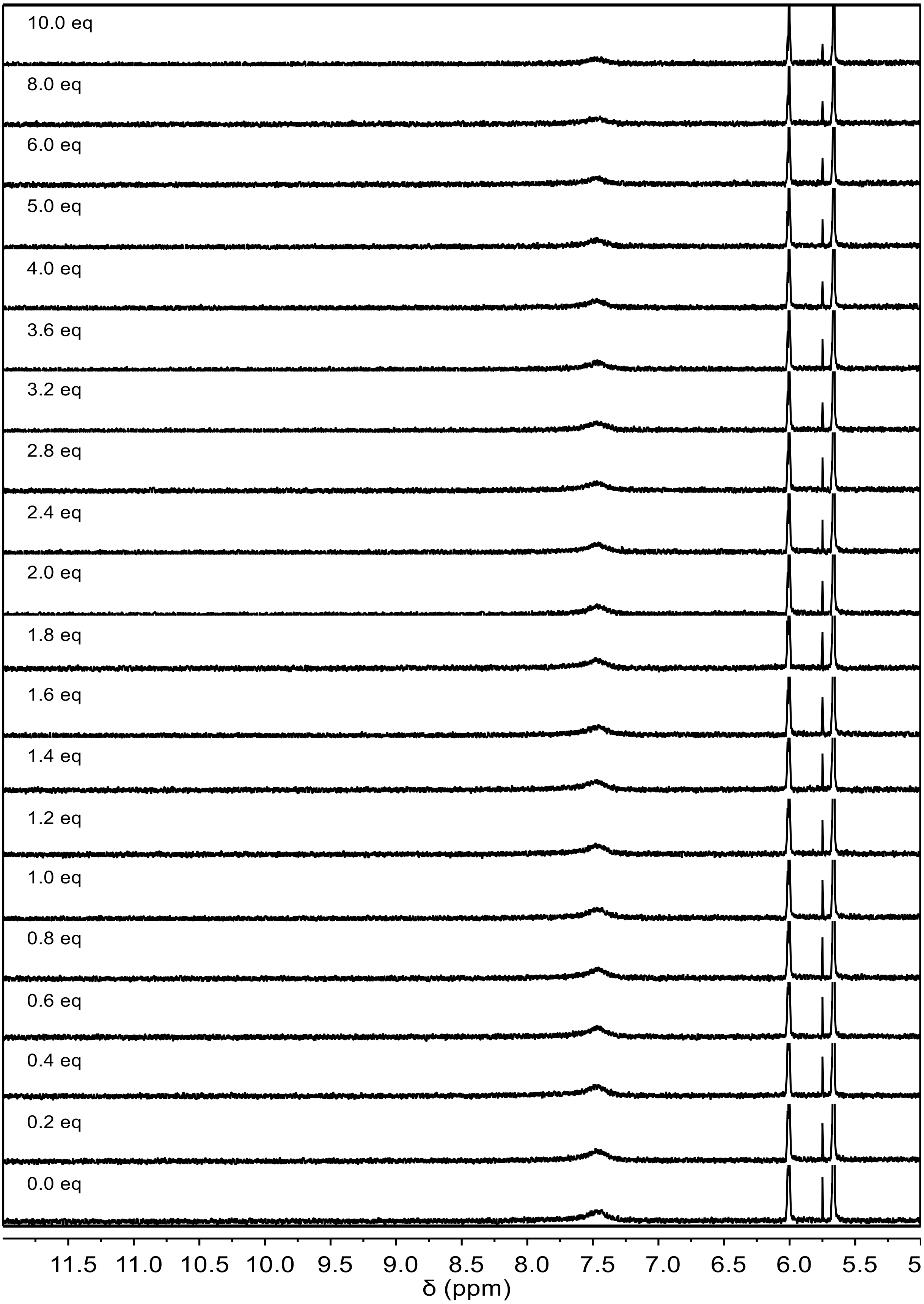

Figure S16. ^{1}H NMR spectral changes in the aromatic region of **SQ2** (DMSO-d_6/0.5%H_2O) upon the stepwise addition of $[Bu_4N]^+[I]^-$.

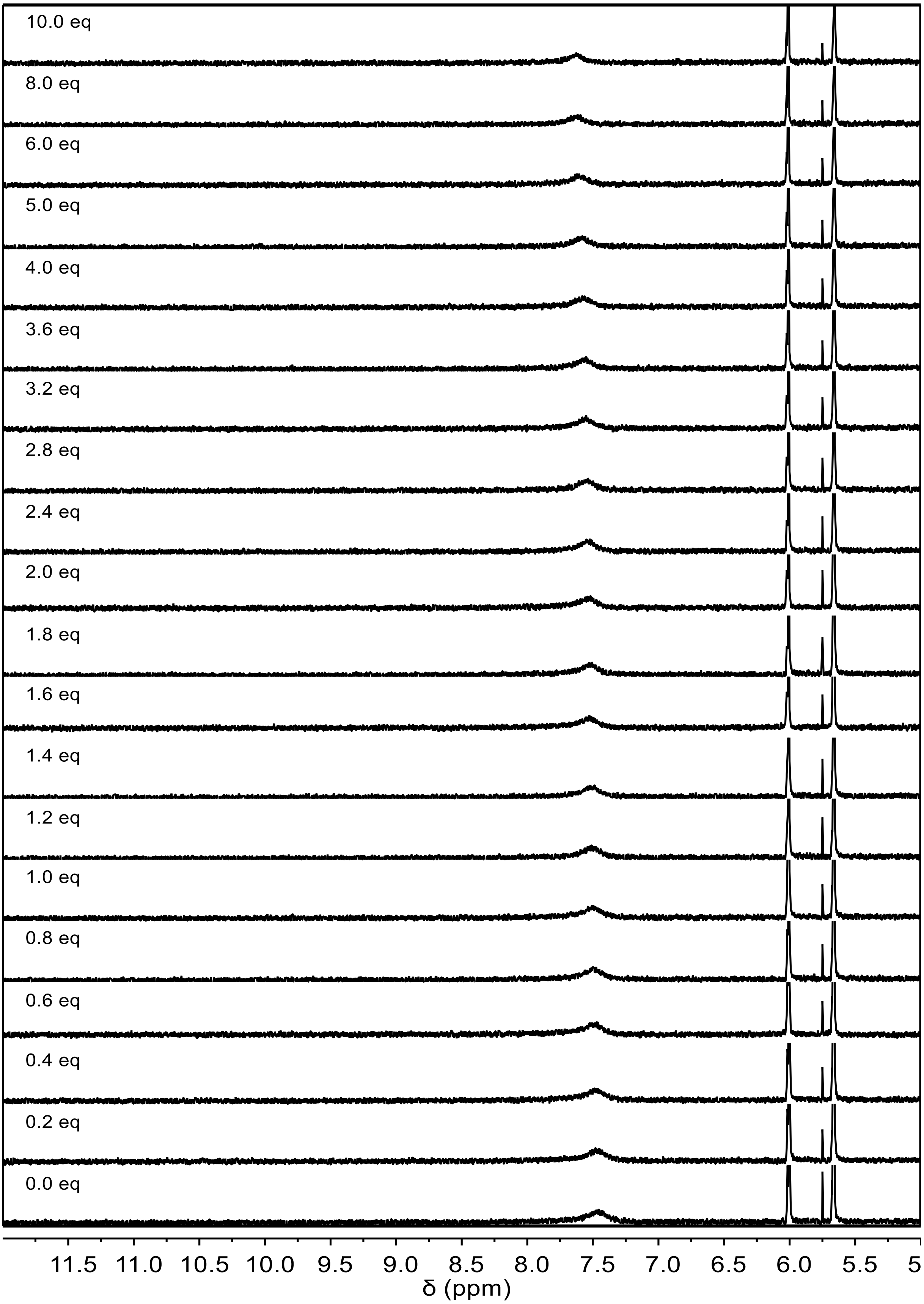

Figure S17. ^{1}H NMR spectral changes in the aromatic region of **SQ2** (DMSO-d_6/0.5%H_2O) upon the stepwise addition of $[Bu_4N]^+[Br]^-$.

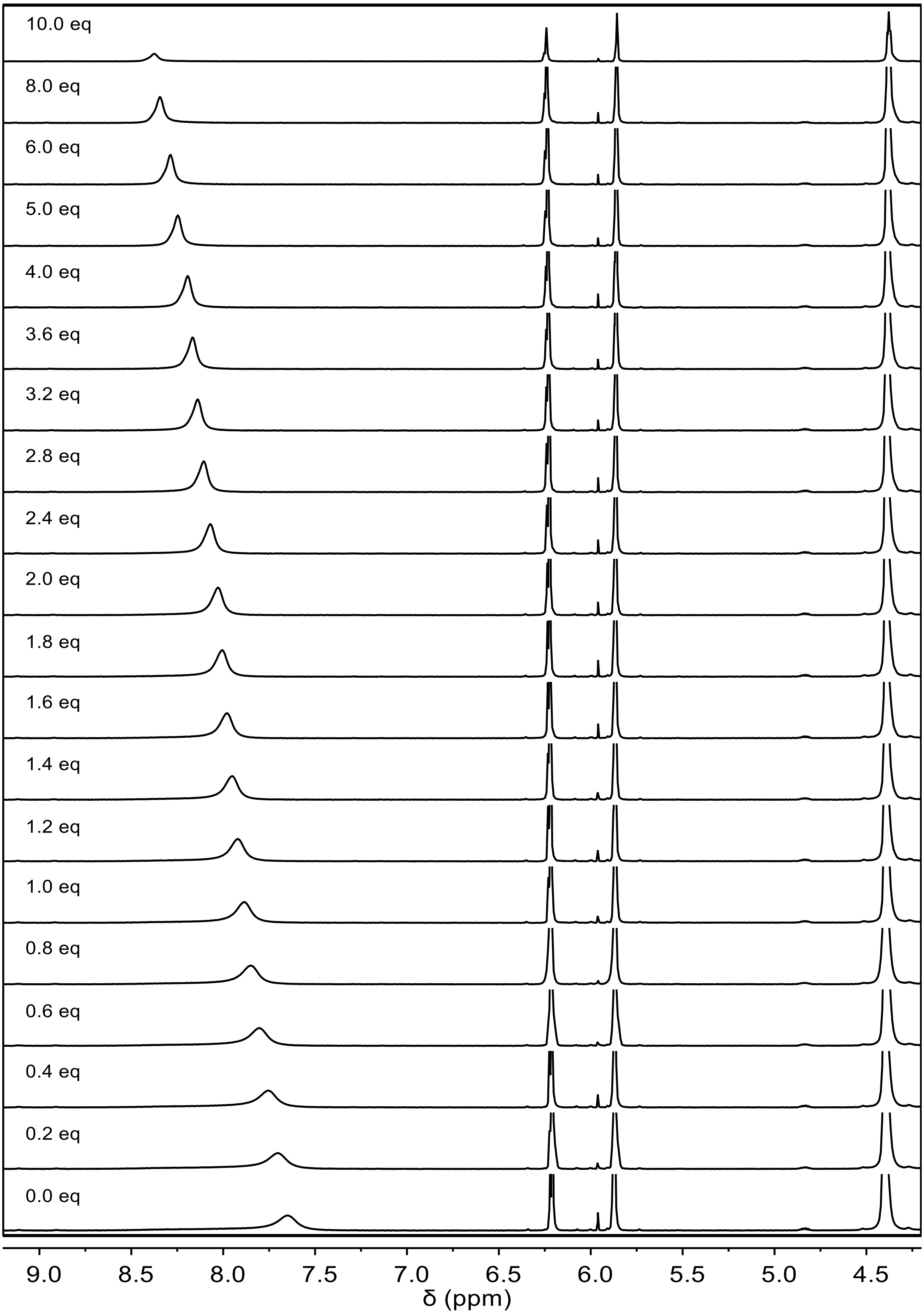

Figure S18. ^{1}H NMR spectral changes in the aromatic region of **SQ2** (DMSO-d_6/0.5%H_2O) upon the stepwise addition of $[Bu_4N]^+[Cl]^-$.

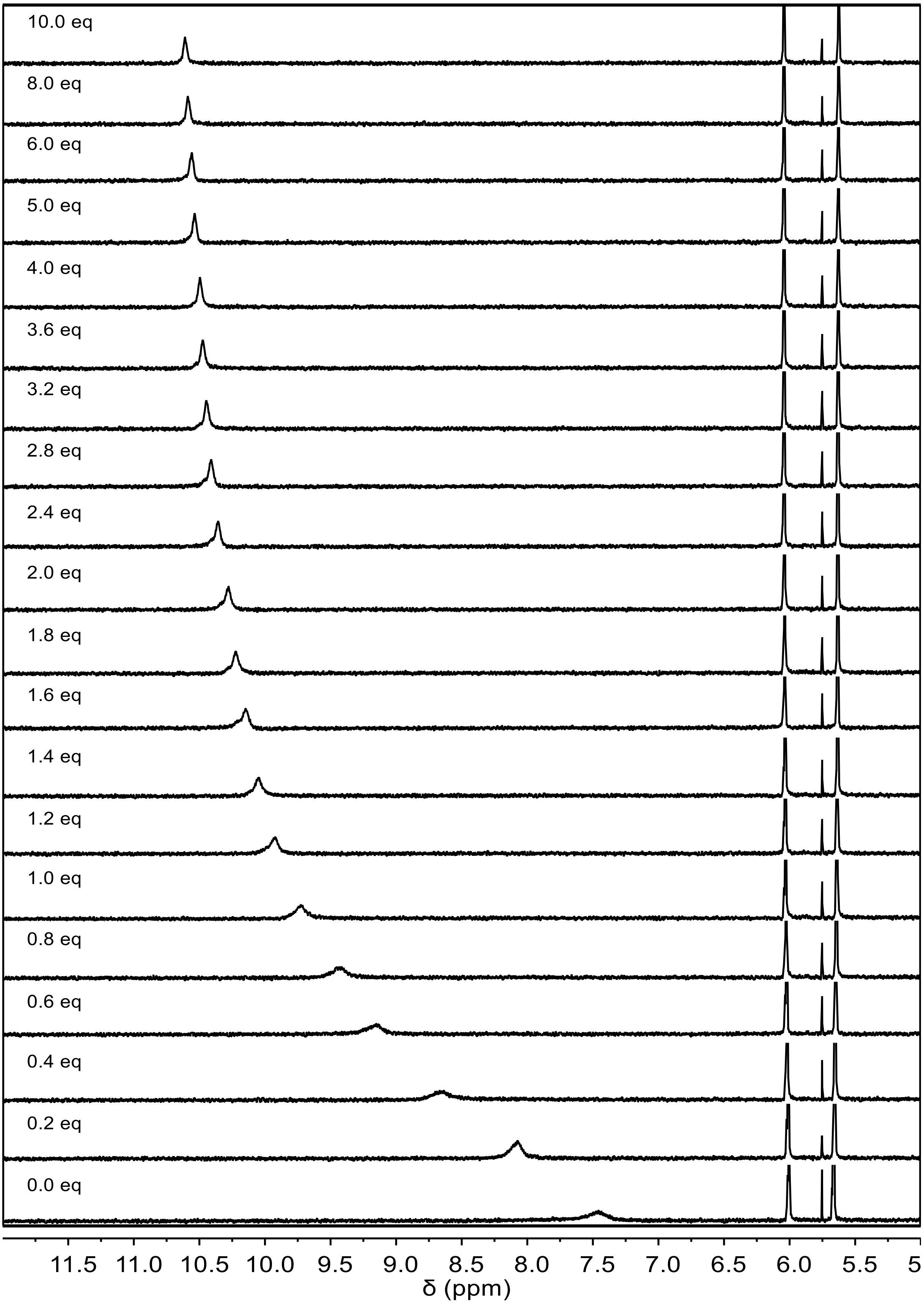

Figure S19. ^{1}H NMR spectral changes in the aromatic region of **SQ2** (DMSO-d_6/0.5%H_2O) upon the stepwise addition of $[Bu_4N]^+[AcO]^-$.

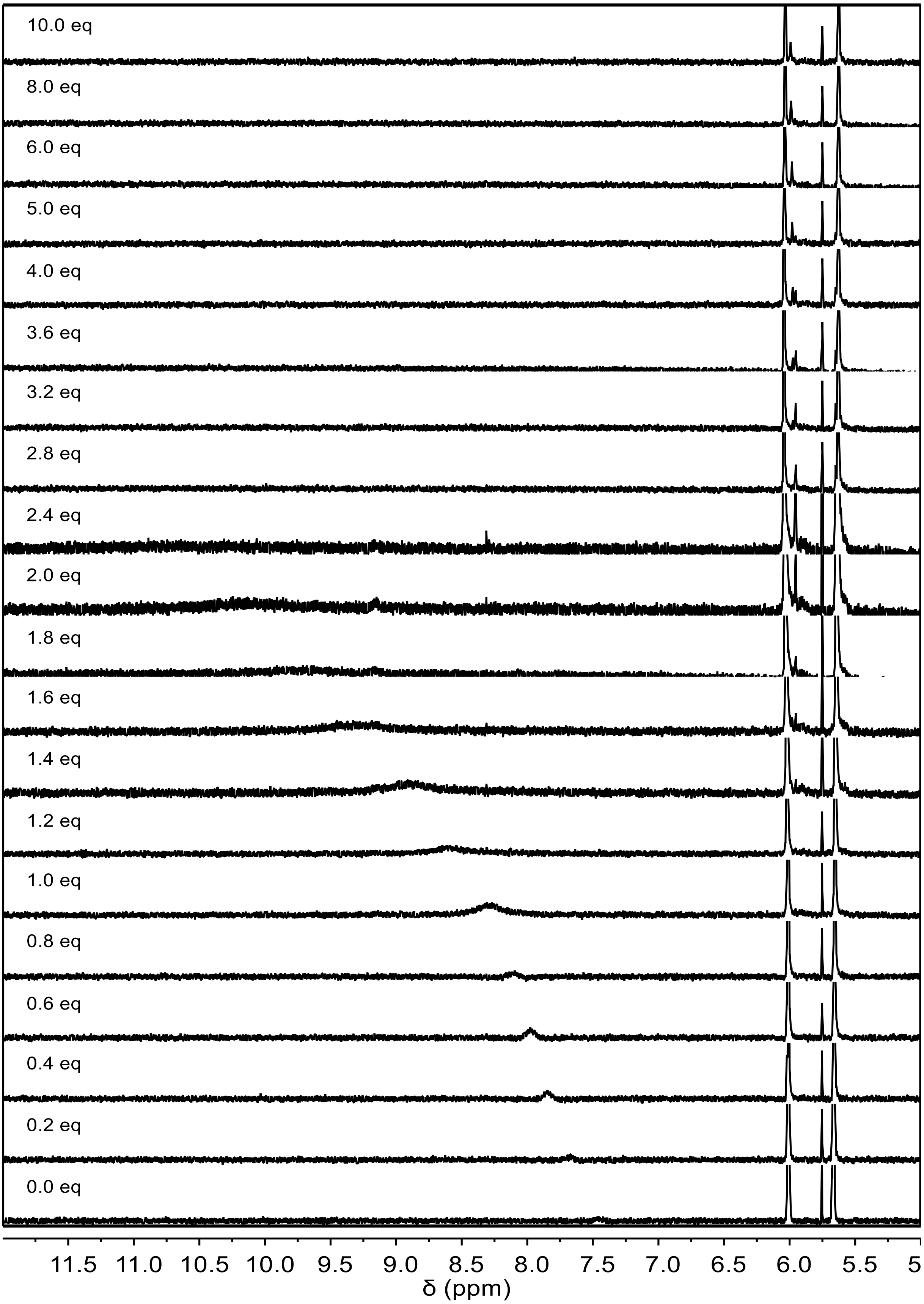

Figure S20. ^{1}H NMR spectral changes in the aromatic region of **SQ2** (DMSO-d_6/0.5%H_2O) upon the stepwise addition of $[Bu_4N]^+[F]^-$.

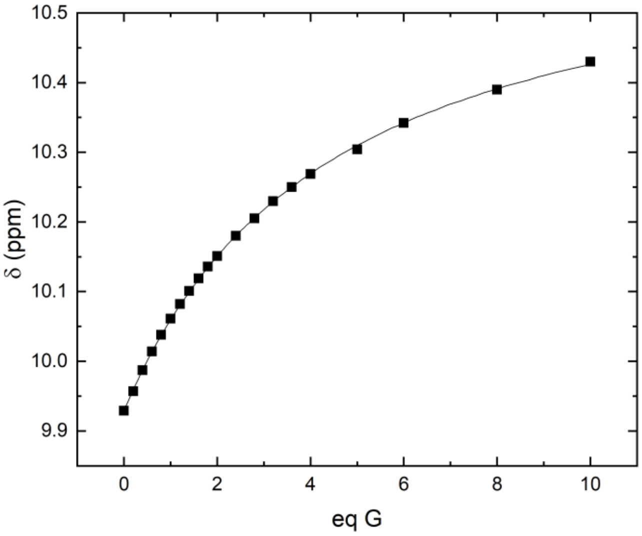

Figure S21. Titration curve generated from addition of $[Bu_4N]^+[Br]^-$ to **SQ1** and data fitting to a 1:1 model obtained by analysis of the squaramide-NH signals: K_a = 31.6 M^{-1}.

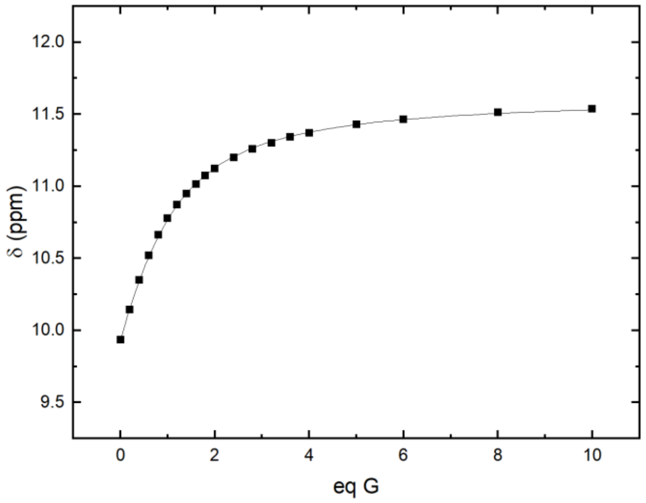

Figure S22. Titration curve generated from addition of $[Bu_4N]^+[Cl]^-$ to **SQ1** and data fitting to a 1:1 model obtained by analysis of the squaramide-NH signals: K_a = 354 M^{-1}.

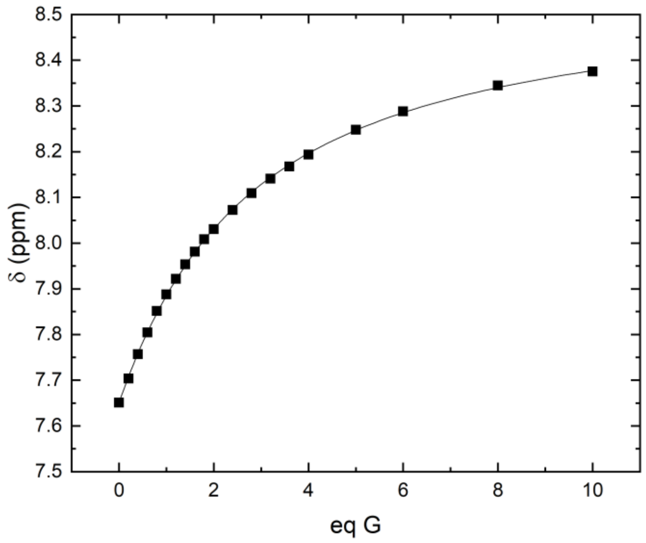

Figure S23. Titration curve generated from addition of $[Bu_4N]^+[Cl]^-$ to **SQ2** and data fitting to a 1:1 model obtained by analysis of the squaramide-NH: K_a = 67.5 M^{-1}.

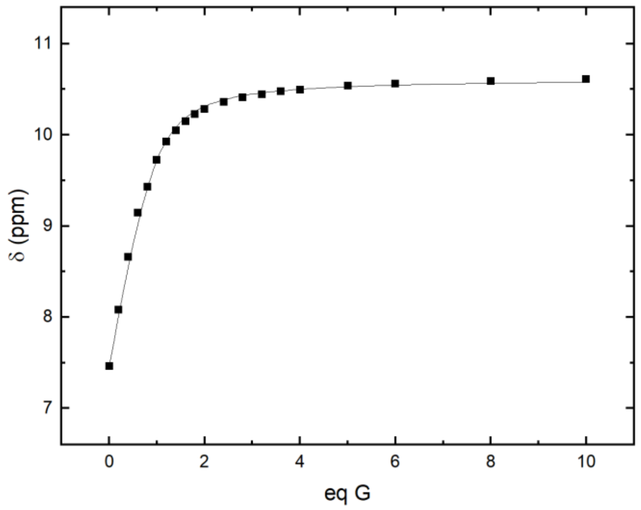

Figure S24. Titration curve generated from addition of $[Bu_4N]^+[AcO]^-$ to **SQ2** and data fitting to a 1:1 model obtained by analysis of the squaramide-NH signals: $K_a = 1.82 \times 10^3$ M^{-1}.

2.2 UV-Vis Titrations

For UV-Vis titrations, 50 mM solutions of receptors (**SQ1** and **SQ2)** and 5 mM solutions of the tetrabutylammonium anion were prepared in a mixture of DMSO and 0.5% H_2O (v/v). Subsequently, the 5 mM anion solution was added stepwise to 2 mL of the 50 mM receptor solution and a UV/Vis spectrum was recorded after each addition. The data was fitted to a 1:1 binding model using the online software BindFit[2-3] with the absorption intensity being observed at a specific wavelength to track host-guest interactions accordingly.

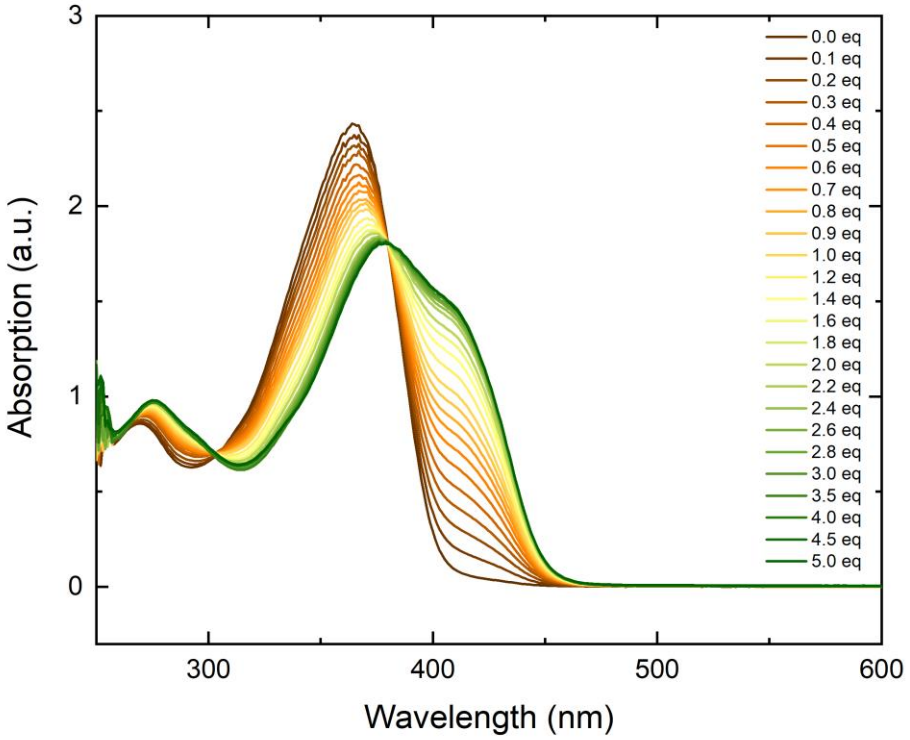

Figure S25. UV/Vis spectral changes of **SQ1** (DMSO/0.5%H_2O) upon the stepwise addition of $[Bu_4N]^+[AcO]^-$.

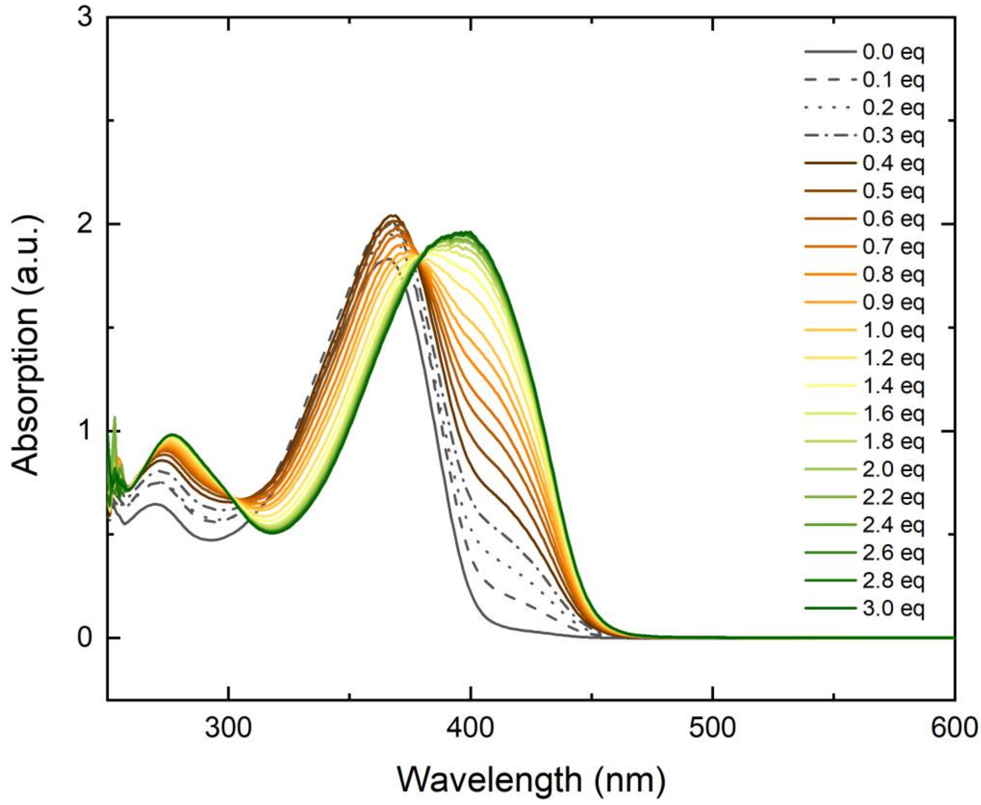

Figure S26. UV/Vis spectral changes of **SQ1** (DMSO/0.5%H_2O) upon the stepwise addition of $[Bu_4N]^+[F]^-$.

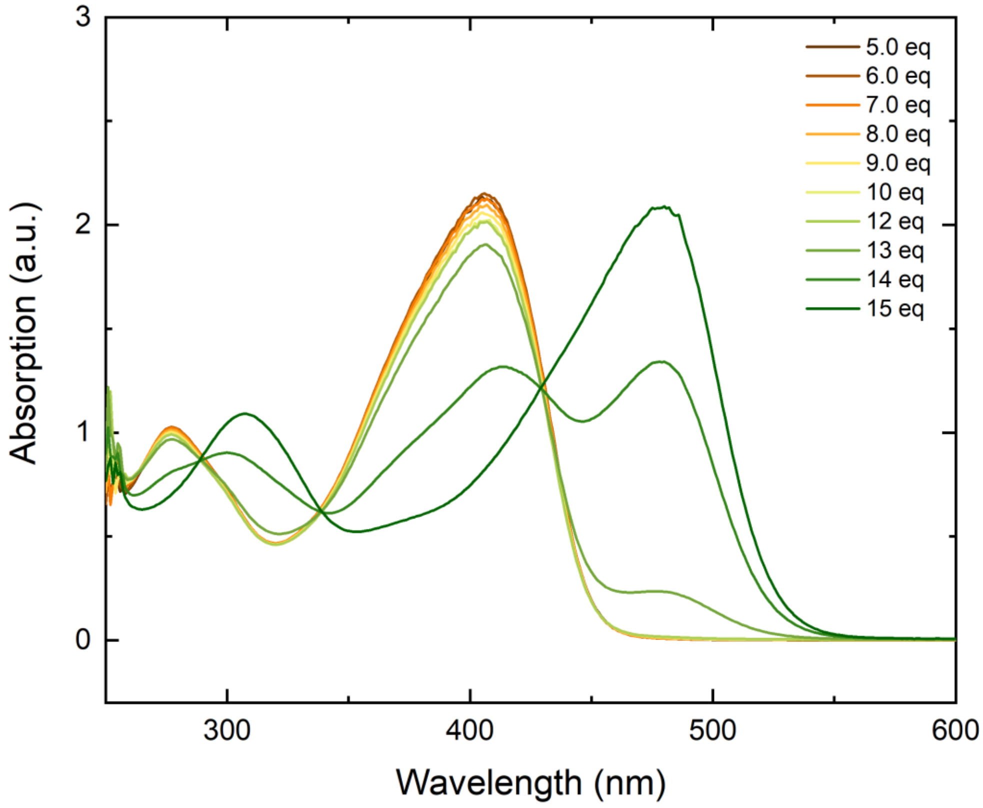

Figure S27. UV/Vis spectral changes of **SQ1** (DMSO/0.5%H_2O) upon the addition of excess $[Bu_4N]^+[OH]^-$.

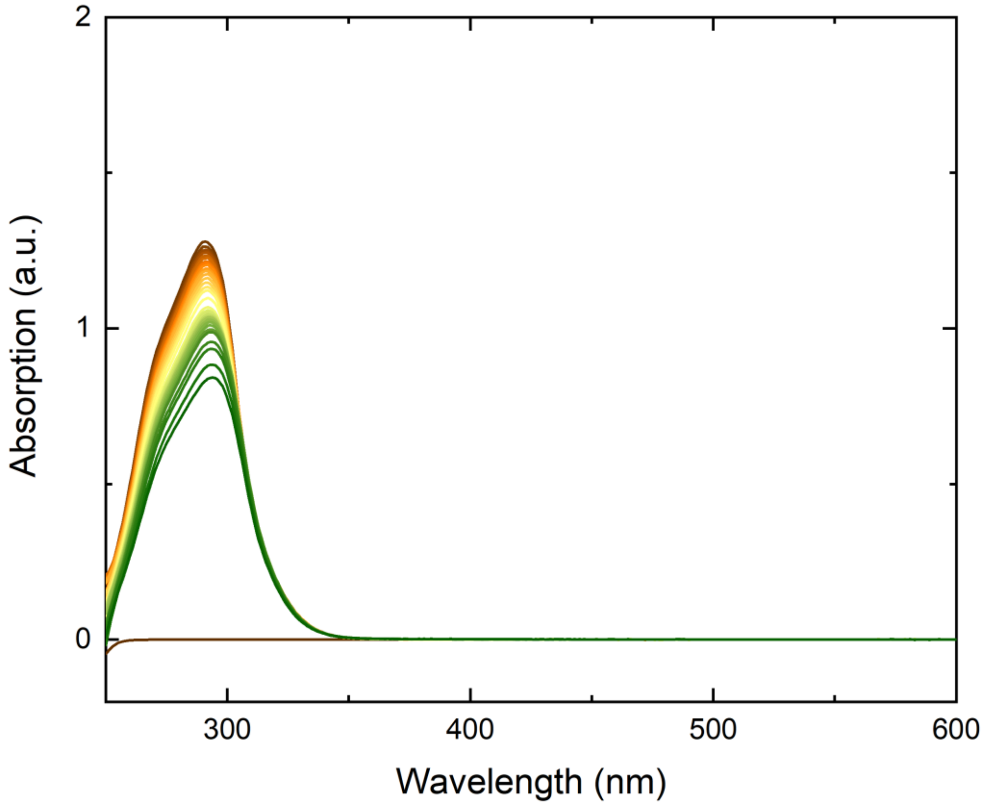

Figure S28. UV/Vis spectral changes of **SQ2** (DMSO/0.5%H_2O) upon the stepwise addition of $[Bu_4N]^+[AcO]^-$.

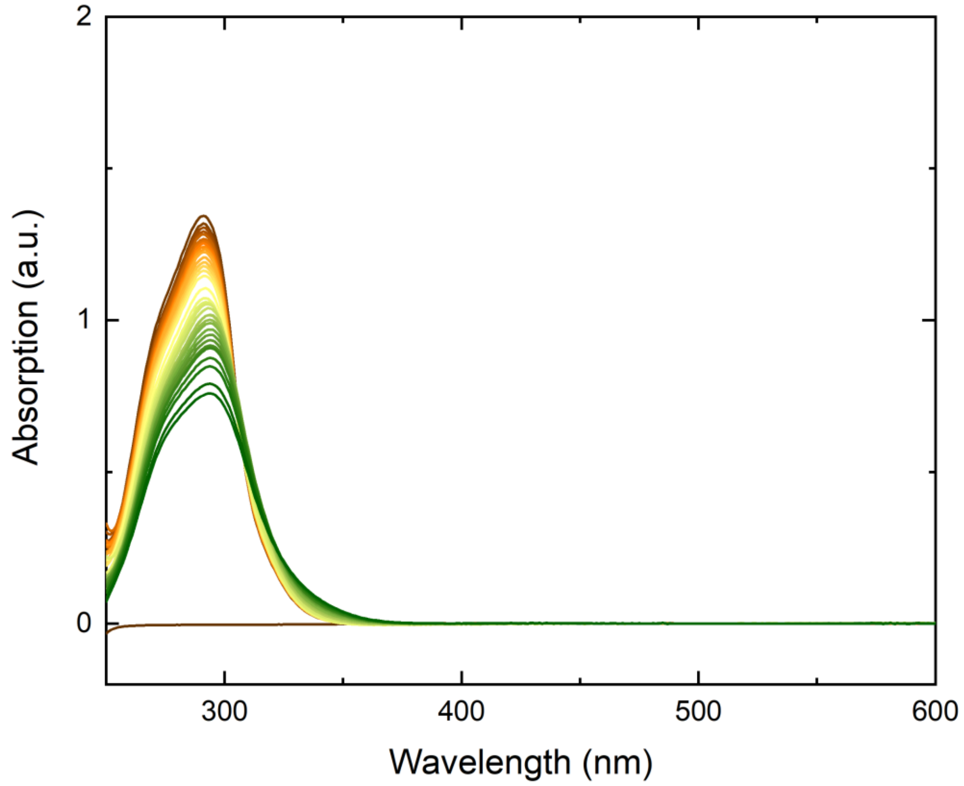

Figure S29. UV/Vis spectral changes of **SQ2** (DMSO/0.5%H_2O) upon the stepwise addition of $[Bu_4N]^+[F]^-$.

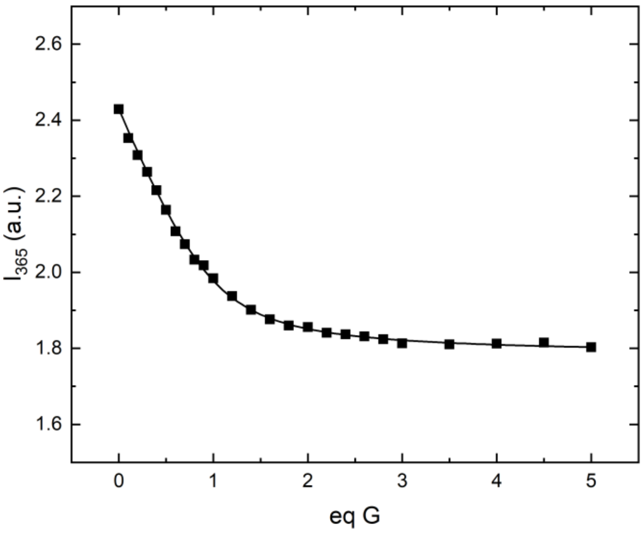

Figure S30. Titration curve generated from addition of $[Bu_4N]^+[AcO]^-$ to **SQ1** and data fitting to a 1:1 model obtained by analysis of the absorption band at 365 nm: $K_a = 7.70 \times 10^4$ M^{-1}.

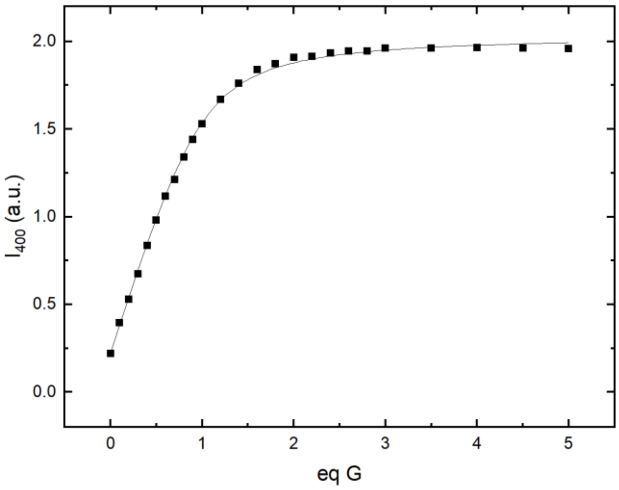

Figure S31. Titration curve generated from the addition of $[Bu_4N]^+[F]^-$ to **SQ1** and data fitting to a 1:1 model obtained by analysis of the absorption band at 400 nm: $K_a = 1.96 \times 10^5$ M^{-1} (slightly lower compared to Manesiotis *et al*. due to the presence of 0.5% H_2O in our case).

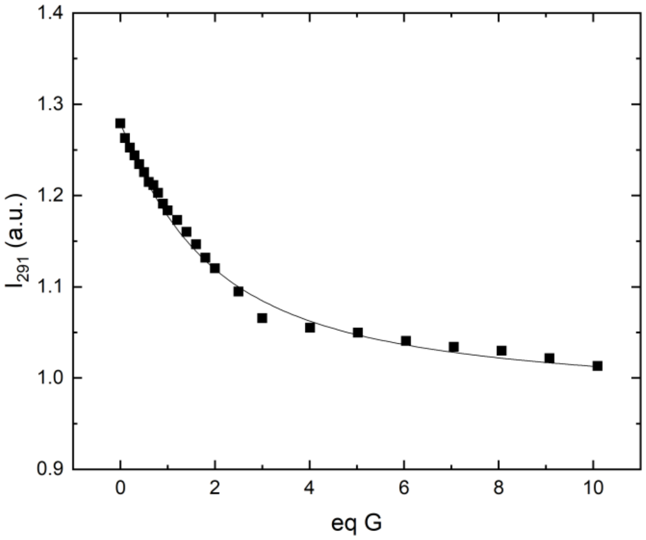

Figure S32. Titration curve generated from addition of $[Bu_4N]^+[AcO]^-$ to **SQ2** and data fitting to a 1:1 model obtained by analysis of the absorption band at 291 nm: $K_a = 1.11 \times 10^3$ M^{-1}.

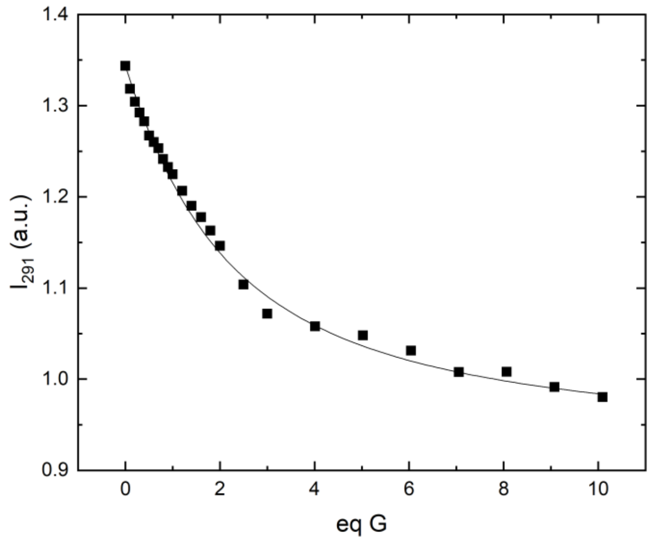

Figure S33. Titration curve generated from addition of $[Bu_4N]^+[F]^-$ to **SQ2** and data fitting to a 1:1 model obtained by analysis of the absorption band at 291 nm: $K_a = 1.03 \times 10^3$ M^{-1}.

3 Gel Characterization

3.1 Equilibrium Swelling

For the swelling experiments, gel specimens were first carefully removed from the glass vials (by breaking them). All gel specimens were weighed prior to the swelling experiment. The weight distribution, average and standard deviation of the **cured gels** were used as measure to assess successful polymerization of each batch and corresponding data is shown as candle stick diagrams (Figure S33-46A). Subsequently, each gel was immersed separately in 3 mL of the corresponding DMSO solution. The highest chosen concentration of $[Bu_4N]^+[X]^-$ salts was set to 1 M. However, due to volume expansion effects during solvation, the apparent concentrations were measured to be lower: $[Bu_4N]^+[I]^-$ (0.767 M), $[Bu_4N]^+[Br]^-$ (0.909 M), $[Bu_4N]^+[Cl]^-$ (0.915 M), $[Bu_4N]^+[AcO]^-$ (0.757 M), $[Bu_4N]^+[F]^-$ (0.810 M). The latter solutions were diluted accordingly to deliver the following concentration row: 0.1 mM, 0.5 mM, 1.0 mM, 5.0 mM, 10 mM, 50 mM, 0.1 M and 0.5 M. Additionally, pure DMSO was used as blank to represent a salt concentration of 0 M. Gels were swollen over the course of 24 h or in chosen cases 7 days, for each concentration in triplicates. After the swelling period, gel specimens were removed from the solution. Gels were gently blotted with small pieces of precision wipes (Kimtech Sciences) to remove excess solvent. Each gel specimen was weighed in the equilibrium swollen state. Next, the gels were shock-frozen using liquid nitrogen prior removal of the solvent *via* lyophilization. The dried gels were then carefully weighed again. The dry weights of gels immersed in 0 mM, 0.1 mM 0.5 mM and 1.0 mM solutions were averaged for the later calculations (4 concentrations in triplicate → 12 values). For concentrations above 1.0 mM, the dry weight started to increase with increasing salt concentration and those samples were therefore not included in the statistics of **dry weights**. The statistics of these data are summarized in Figures S33-46B.

3.2 Mass & Volume Swelling Ratio

The mass and volume swelling ratios were calculated according to formulas used in the Flor-Rehner mesh size theory.[4-5] Accordingly, the mass swelling ratio Q_m is defined as the ratio of m_s, the gel mass in equilibrium swollen state, and the dry mass m_d.

$$Q_m = \frac{m_s}{m_d} \tag{S1}$$

Next, the mass swelling ratio Q_m is used to calculate the volume swelling ratio Q_V (equation S2) where ρ_p and ρ_s are defined as the density of the solid polymer and the solvent (water), respectively.

$$Q_V = 1 + \frac{\rho_P}{\rho_S}(Q_m - 1) \tag{S2}$$

The density of DMSO is ρ_s = 1.1 g/cm³ and the density of poly(*N*,*N*-dimethylacrylamide) was approximated to ρ_p = 1.213 g/cm³.[6]

3.3 Supporting Swelling Experiments

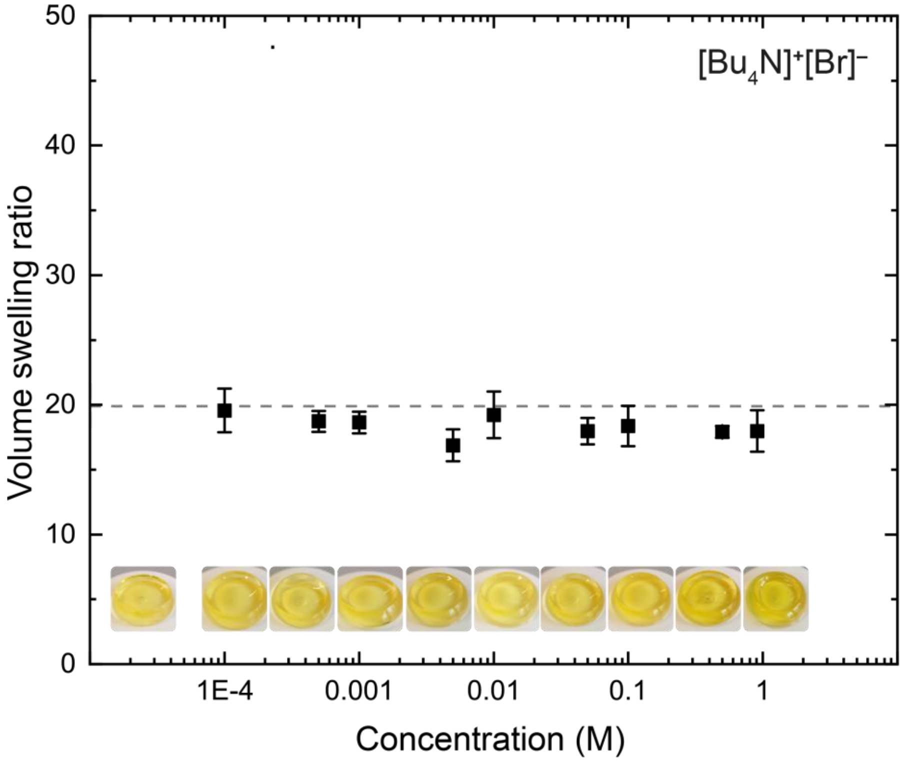

Figure S34. Volume swelling ratios of **SQ1**-crosslinked gels vs. concentration of $[Bu_4N]^+[Br]^-$ after 1 d (black squares, n=3). Dashed line represents swelling in pure DMSO. Pictures from left to right: 0, 0.1, 0.5, 1, 5, 10, 50, 100, 500, 910 mM.

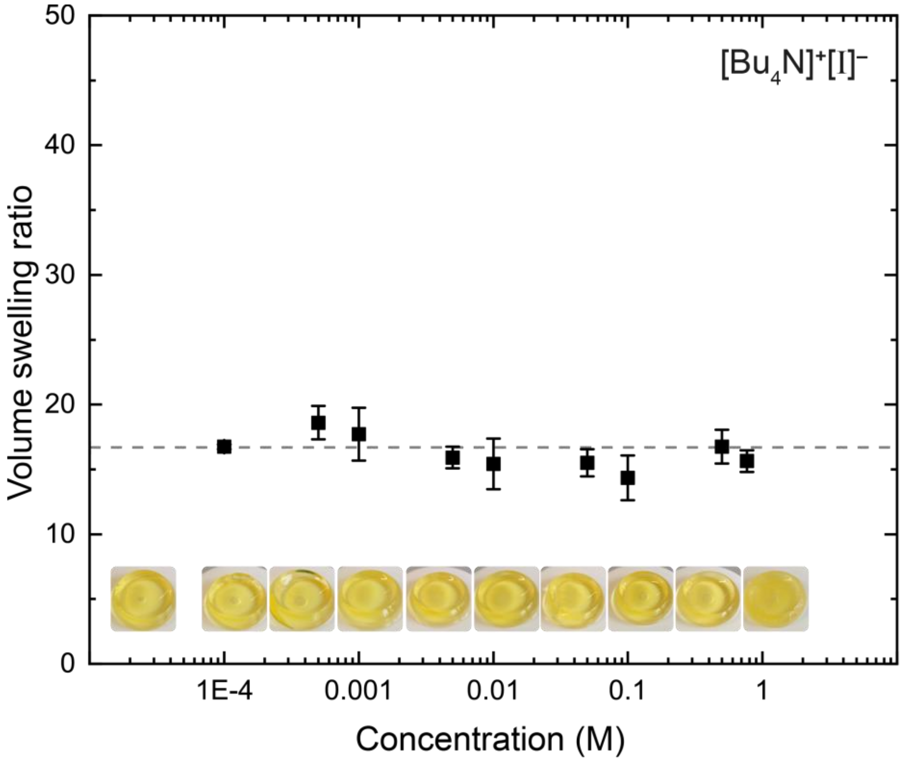

Figure S35. Volume swelling ratios of **SQ1**-crosslinked gels vs. concentration of $[Bu_4N]^+[I]^-$ after 1 d (black squares, n=3). Dashed line represents swelling in pure DMSO. Pictures from left to right: 0, 0.1, 0.5, 1, 5, 10, 50, 100, 500, 780 mM.

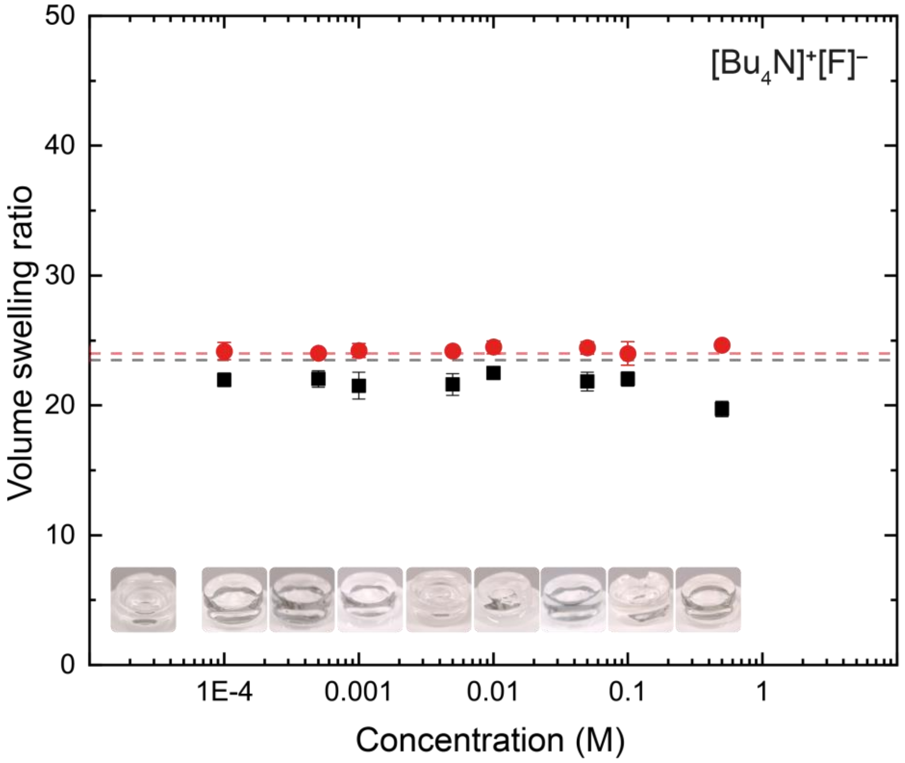

Figure S36. Volume swelling ratios (n=3) of EGDMA-crosslinked gels vs. concentration of $[Bu_4N]^+[F]^-$ after 1 d (black squares) and 7 d (red spheres). Dashed lines represent swelling in pure DMSO. Pictures from left to right: 0, 0.1, 0.5, 1, 5, 10, 50, 100, 500 mM.

3.4 Gravimetric statistics on gel samples

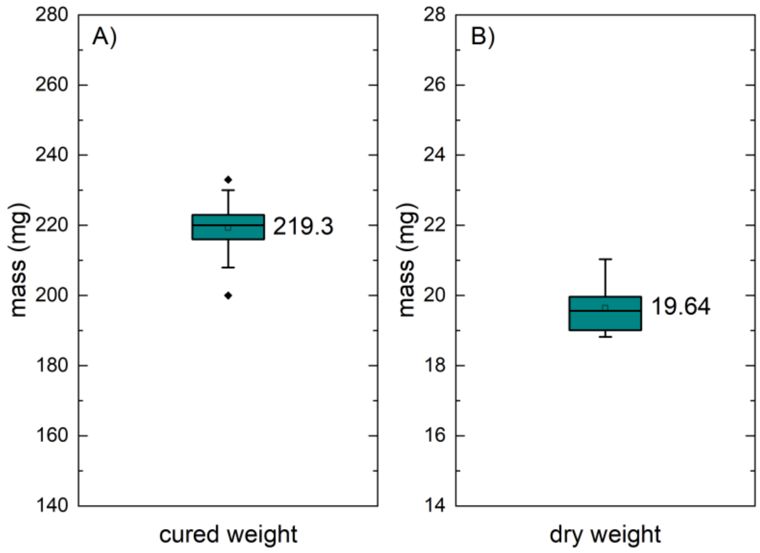

Figure S37. Box and Whiskers plot showing interquartile ranges (25,75 percentiles) with median line (50%), whiskers (5,95 range), outliers (black diamonds) and arithmetic mean (hollow square and label) of A) cured weights and B) dry weights of **SQ1**-crosslinked gels for the 1 d swelling experiment in $[Bu_4N]^+[Cl]^-$.

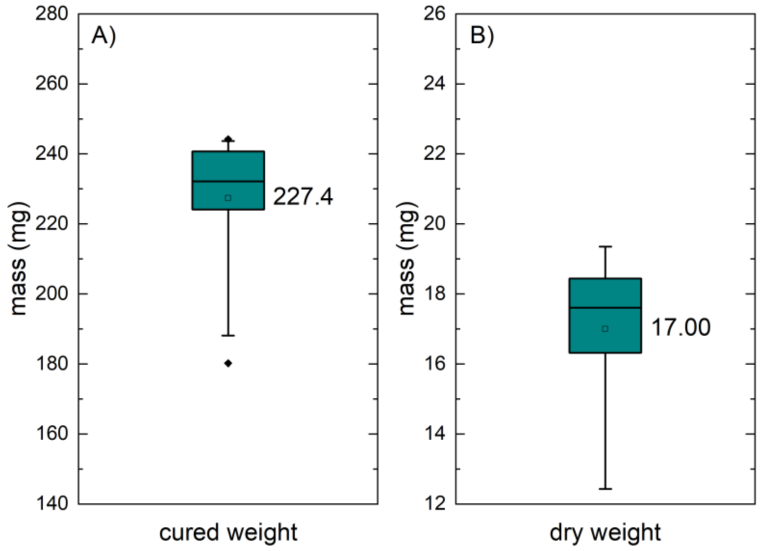

Figure S38. Box and Whiskers plot showing interquartile ranges (25,75 percentiles) with median line (50%), whiskers (5,95 range), outliers (black diamonds) and arithmetic mean (hollow square and label) of A) cured weights and B) dry weights of **SQ1**-crosslinked gels for the 7 d swelling experiment in $[Bu_4N]^+[Cl]^-$.

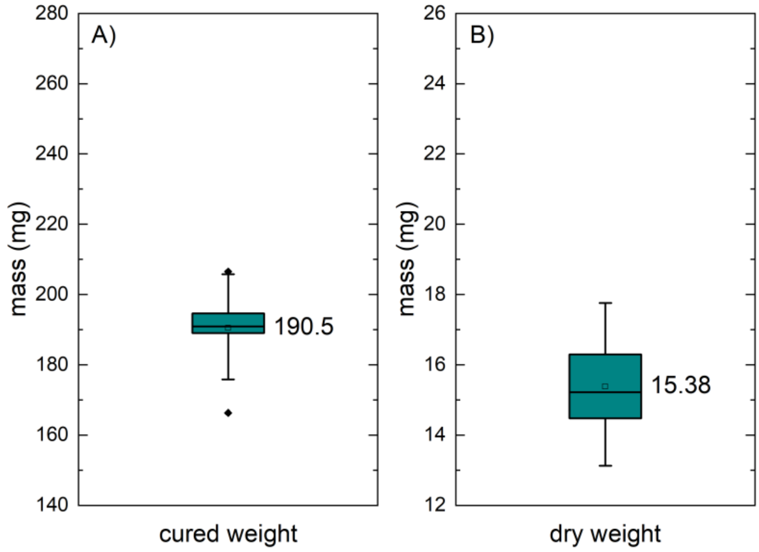

Figure S39. Box and Whiskers plot showing interquartile ranges (25,75 percentiles) with median line (50%), whiskers (5,95 range), outliers (black diamonds) and arithmetic mean (hollow square and label) of A) cured weights and B) dry weights of **SQ1**-crosslinked gels for the 1 d swelling experiment in $[Bu_4N]^+[AcO]^-$.

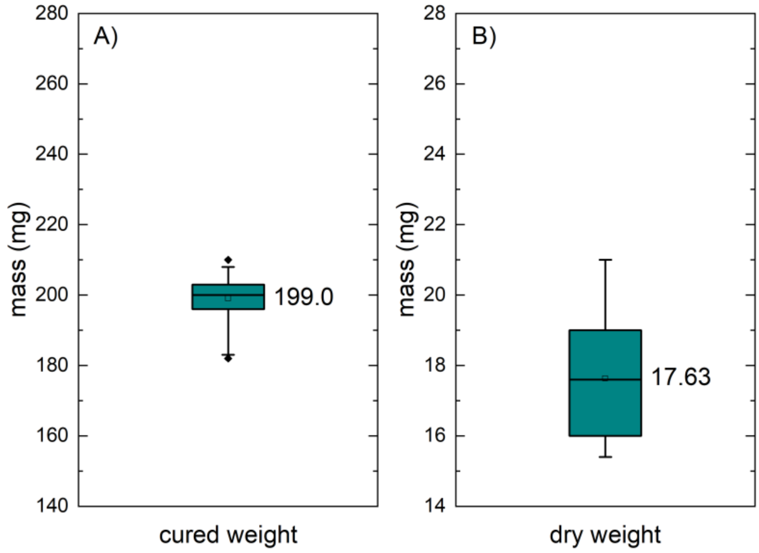

Figure S40. Box and Whiskers plot showing interquartile ranges (25,75 percentiles) with median line (50%), whiskers (5,95 range), outliers (black diamonds) and arithmetic mean (hollow square and label) of A) cured weights and B) dry weights of **SQ1**-crosslinked gels for the 7 d swelling experiment in $[Bu_4N]^+[AcO]^-$.

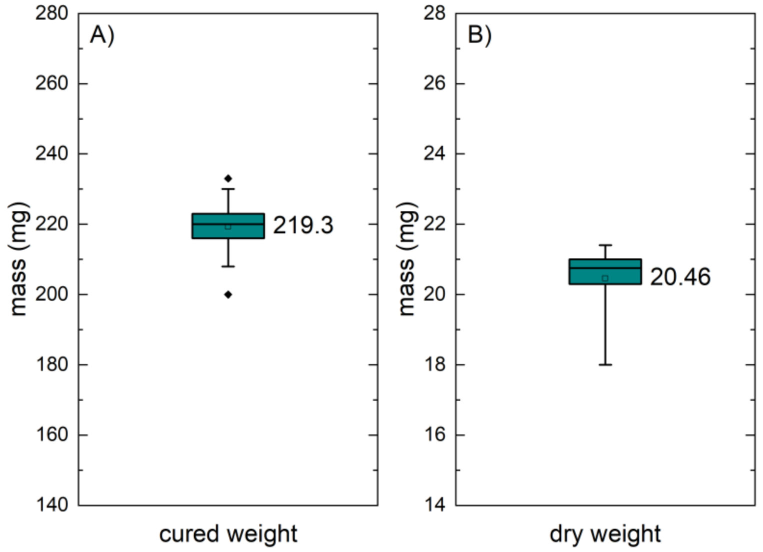

Figure S41. Box and Whiskers plot showing interquartile ranges (25,75 percentiles) with median line (50%), whiskers (5,95 range), outliers (black diamonds) and arithmetic mean (hollow square and label) of A) cured weights and B) dry weights of **SQ1**-crosslinked gels for the 1 d swelling experiment in $[Bu_4N]^+[F]^-$.

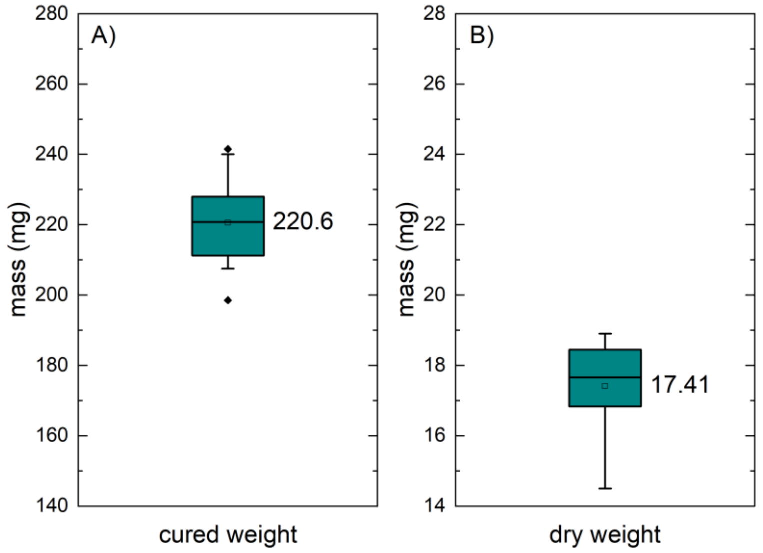

Figure S42. Box and Whiskers plot showing interquartile ranges (25,75 percentiles) with median line (50%), whiskers (5,95 range), outliers (black diamonds) and arithmetic mean (hollow square and label) of A) cured weights and B) dry weights of **SQ1**-crosslinked gels for the 7 d swelling experiment in $[Bu_4N]^+[F]^-$.

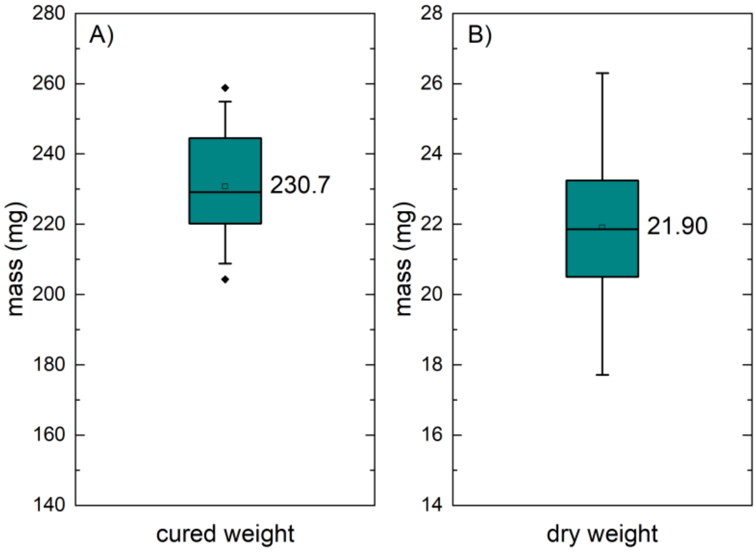

Figure S43. Box and Whiskers plot showing interquartile ranges (25,75 percentiles) with median line (50%), whiskers (5,95 range), outliers (black diamonds) and arithmetic mean (hollow square and label) of A) cured weights and B) dry weights of **SQ1**-crosslinked gels for the 1 d swelling experiment in $[Bu_4N]^+[I]^-$.

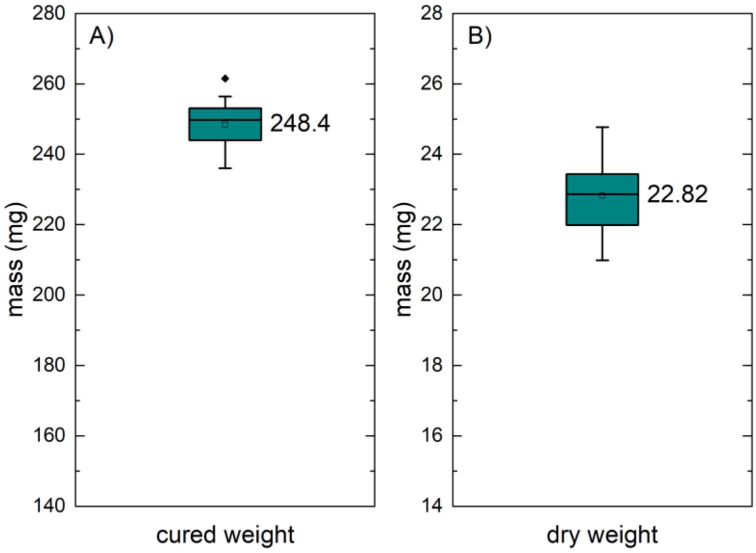

Figure S44. Box and Whiskers plot showing interquartile ranges (25,75 percentiles) with median line (50%), whiskers (5,95 range), outliers (black diamonds) and arithmetic mean (hollow square and label) of A) cured weights and B) dry weights of **SQ1**-crosslinked gels for the 1 d swelling experiment in $[Bu_4N]^+[Br]^-$.

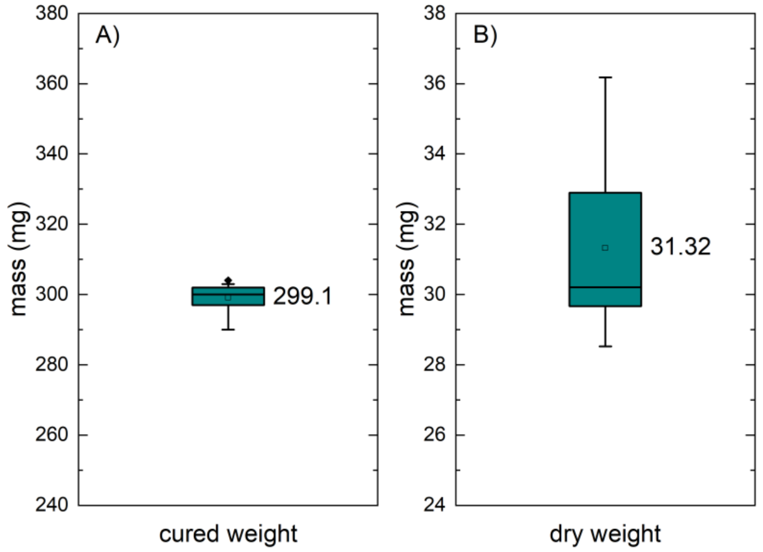

Figure S45. Box and Whiskers plot showing interquartile ranges (25,75 percentiles) with median line (50%), whiskers (5,95 range), outliers (black diamonds) and arithmetic mean (hollow square and label) of A) cured weights and B) dry weights of **SQ2**-crosslinked gels for the 1 d swelling experiment in $[Bu_4N]^+[Cl]^-$.

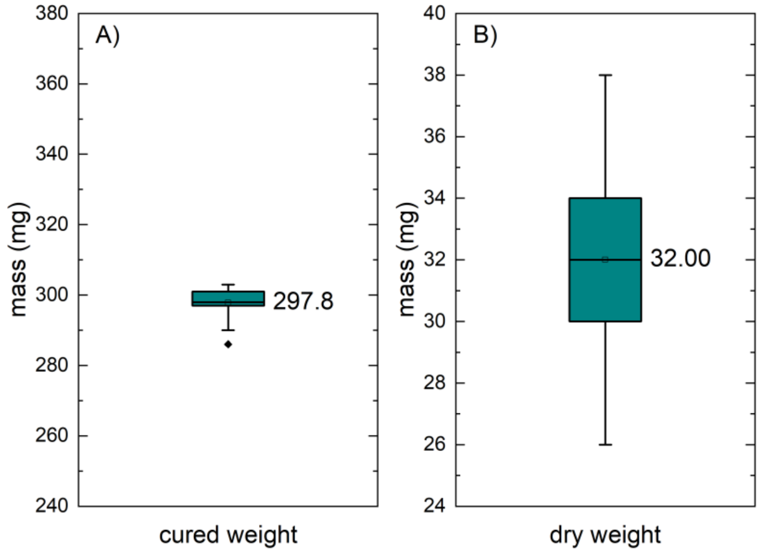

Figure S46. Box and Whiskers plot showing interquartile ranges (25,75 percentiles) with median line (50%), whiskers (5,95 range), outliers (black diamonds) and arithmetic mean (hollow square and label) of A) cured weights and B) dry weights of **SQ2**-crosslinked gels for the 7 d swelling experiment in $[Bu_4N]^+[Cl]^-$.

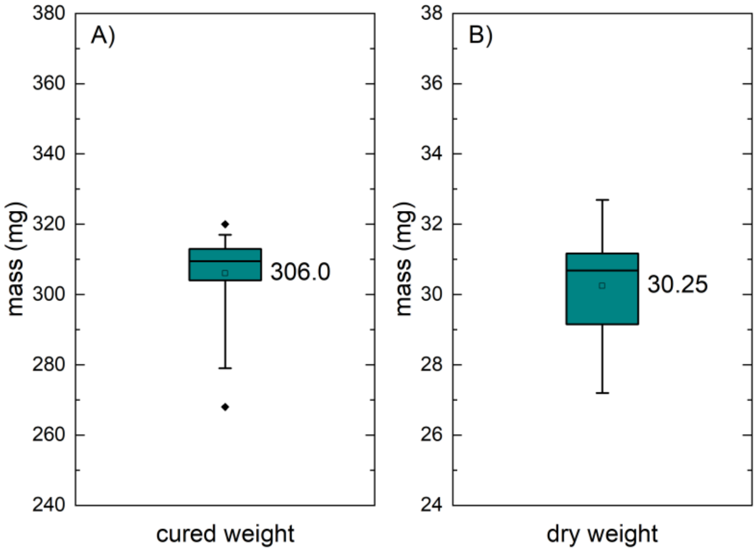

Figure S47. Box and Whiskers plot showing interquartile ranges (25,75 percentiles) with median line (50%), whiskers (5,95 range), outliers (black diamonds) and arithmetic mean (hollow square and label) of A) cured weights and B) dry weights of **SQ2**-crosslinked gels for the 1 d swelling experiment in $[Bu_4N]^+[AcO]^-$.

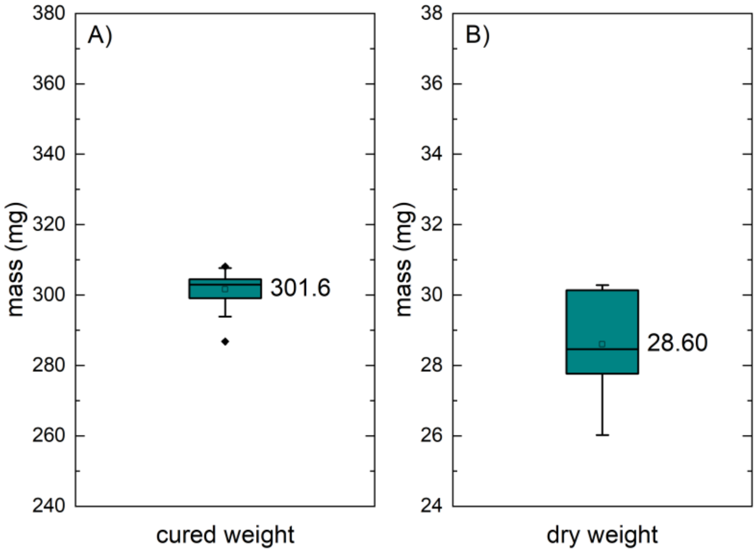

Figure S48. Box and Whiskers plot showing interquartile ranges (25,75 percentiles) with median line (50%), whiskers (5,95 range), outliers (black diamonds) and arithmetic mean (hollow square and label) of A) cured weights and B) dry weights of **SQ2**-crosslinked gels for the 7 d swelling experiment in $[Bu_4N]^+[AcO]^-$.

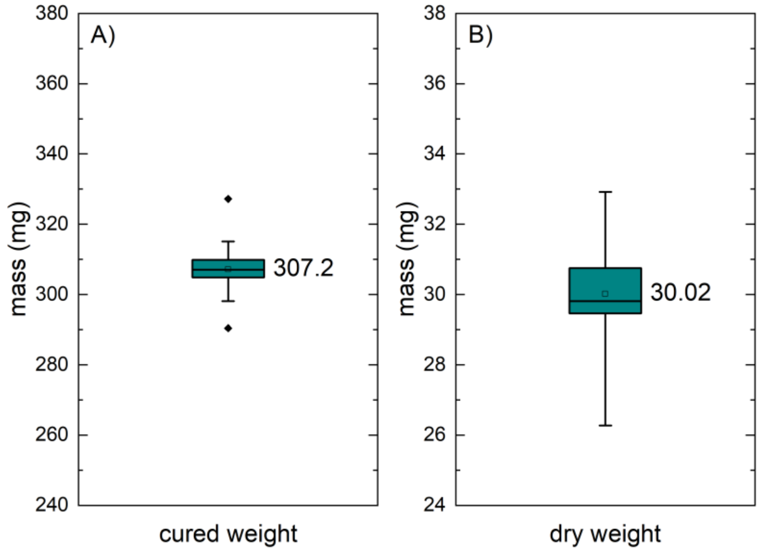

Figure S49. Box and Whiskers plot showing interquartile ranges (25,75 percentiles) with median line (50%), whiskers (5,95 range), outliers (black diamonds) and arithmetic mean (hollow square and label) of A) cured weights and B) dry weights of **SQ2**-crosslinked gels for the 1 d swelling experiment in $[Bu_4N]^+[F]^-$.

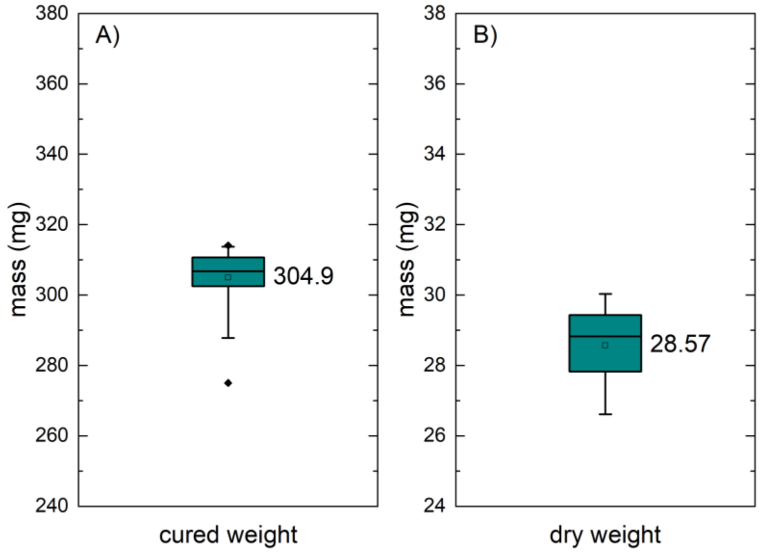

Figure S50. Box and Whiskers plot showing interquartile ranges (25,75 percentiles) with median line (50%), whiskers (5,95 range), outliers (black diamonds) and arithmetic mean (hollow square and label) of A) cured weights and B) dry weights of **SQ2**-crosslinked gels for the 7 d swelling experiment in $[Bu_4N]^+[F]^-$.

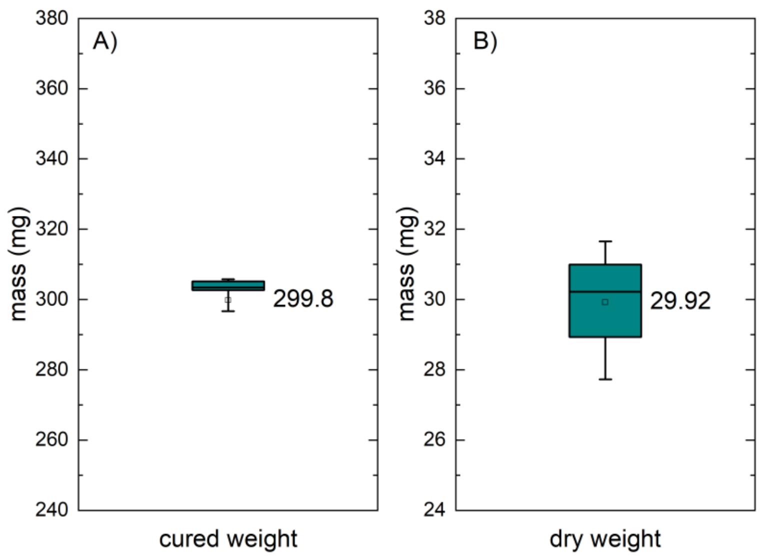

Figure S51. Box and Whiskers plot showing interquartile ranges (25,75 percentiles) with median line (50%), whiskers (5,95 range), outliers (black diamonds) and arithmetic mean (hollow square and label) of A) cured weights and B) dry weights of EGDMA-crosslinked gels for the swelling experiment in $[Bu_4N]^+[F]^-$.

3.5 Rheology

For the rheological measurements, gels were prepared in glass vials as previously described. Three different concentrations of $[Bu_4N]^+[F]^-$ in DMSO were prepared (0 M, 10 mM and 0.8 M). Each gel sample was then immersed in the respective solution for 24 h. Next, the gel was removed and cut with a razorblade to obtain thin gel slices that were then rapidly loaded onto the rheometer.

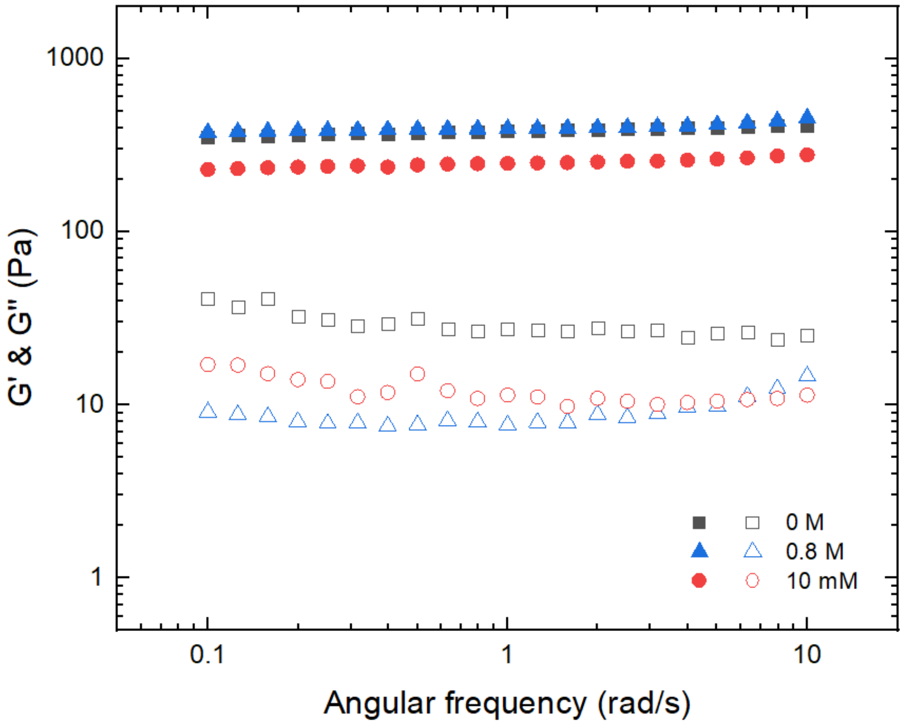

Figure S52. Frequency dependent oscillatory rheology (g = 1%) of **SQ1**-crosslinked polymer networks after swelling in 0 M, 10 mM and 0.8 M $[Bu_4N]^+[F]^-$ DMSO solutions for 24 h.

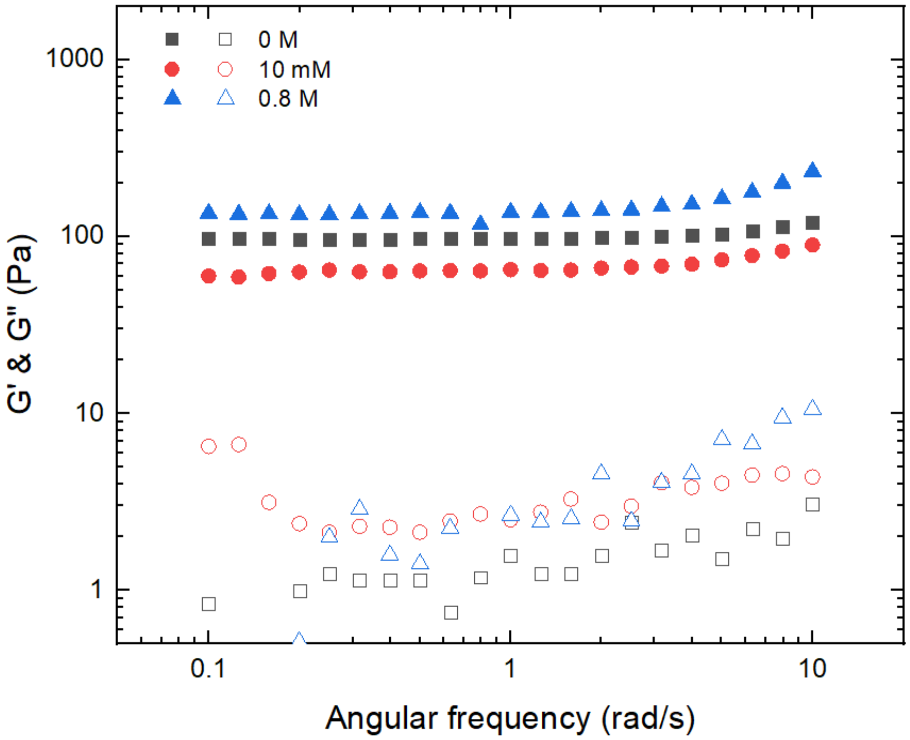

Figure S53. Frequency dependent oscillatory rheology (g = 1%) of **SQ2**-crosslinked polymer networks after swelling in 0 M, 10 mM and 0.8 M $[Bu_4N]^+[F]^-$ DMSO solutions for 24 h.

4 Cycling Swelling Experiments

For the cycling swelling experiments, gel specimens were first carefully removed from the glass vials (by breaking them). Subsequently, each gel was immersed separately in 3 mL of the corresponding DMSO salt solution. The highest chosen concentration of $[Bu_4N]^+[X]^-$ was set to 1 M. However, due to volume expansion effects during solvation, the apparent concentrations were measured to be lower: $[Bu_4N]^+[AcO]^-$ (0.757 M), $[Bu_4N]^+[F]^-$ (0.810 M). Gels were swollen over the course of 24 h or as in Figure S51 7 days. After the swelling period, gel specimens were removed from the solution. Gels were gently blotted with small pieces of precision wipes (Kimtech Sciences) to remove excess solvent. Each gel specimen was weighed in the equilibrium swollen state. Next, the gels were immersed in pure DMSO over the course of 24 h or as in Figure S51 7 days. After the swelling period, gel specimens were removed from the solutions, gently blotted again, and weighed to obtain the next value in the cycling experiment. At the end of the measurement row the gels were shock-frozen using liquid nitrogen prior removal of the solvent *via* lyophilization. The dried gels were then carefully weighed again to determine the volume swelling ratio.

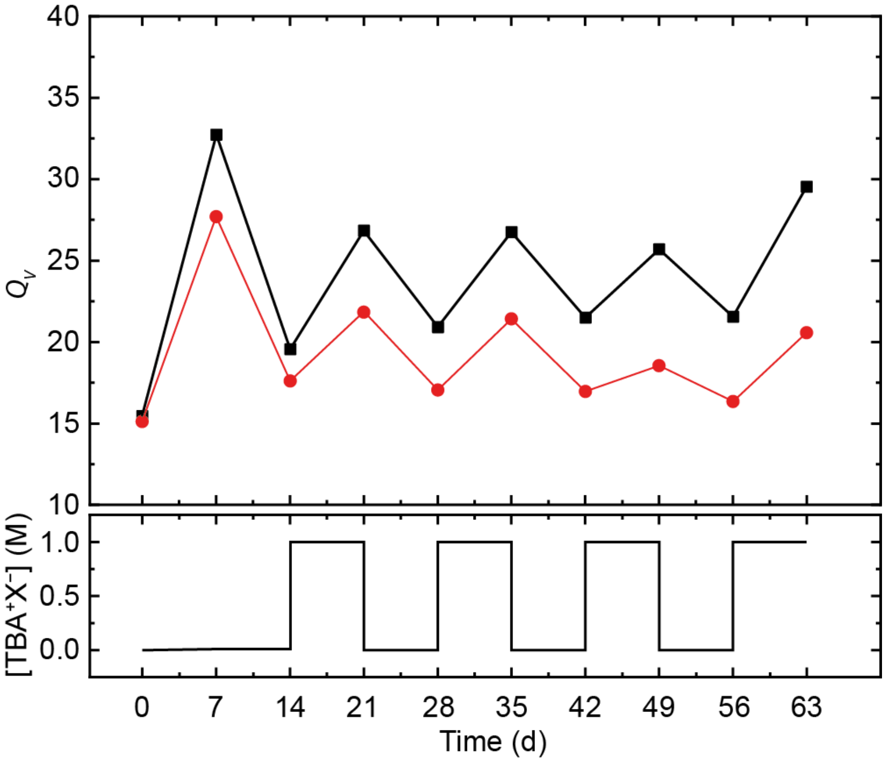

Figure S54. Volume swelling ratios of **SQ1**-crosslinked gel sample after synthesis (day 0), immersed in a 10 mM $[Bu_4N]^+[X]^-$ DMSO solution (day 7) and periodically swollen/collapsed in 0.8 M and 0 M $[Bu_4N]^+[X]^-$ DMSO solutions, respectively (day 14-63). Color code: $[Bu_4N]^+[F]^-$ (black squares), $[Bu_4N]^+[AcO]^-$ (red spheres).

5 Rod-like Gels using a PEEK Mold

A PEEK-based mold was created to prepare rod-shaped gel samples (Figure S50). The mold consisted of two chambers that remained opened towards the flat PEEK mold surface (approximate dimensions L × W × H: 30 mm × 5 mm × 5 mm). On top of the mold, a circular cutout was located that fitted an NS14 rubber seal. The upper side of each chamber was connected via a short tubular channel with the top opening.

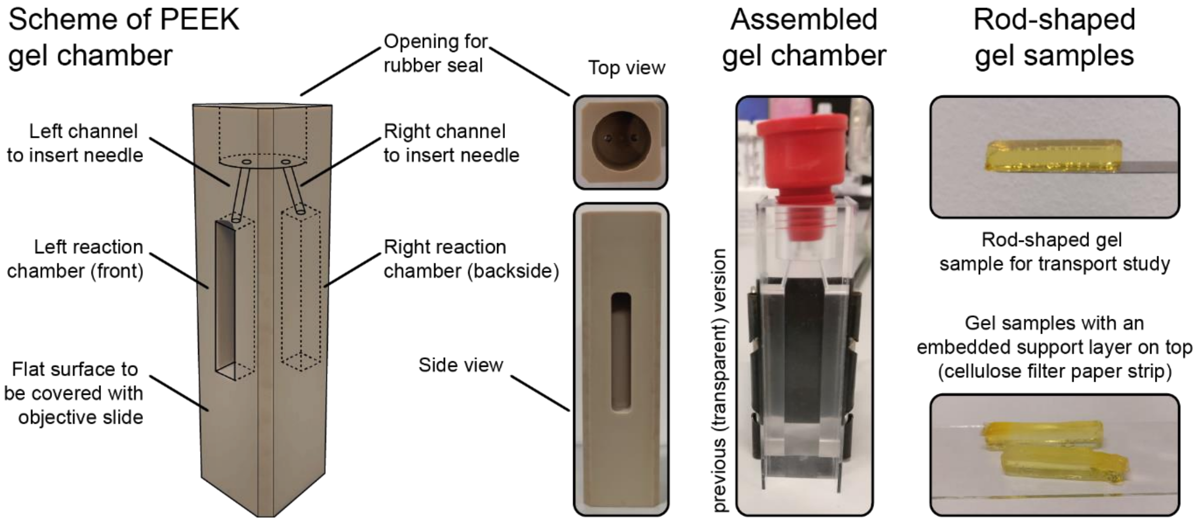

Figure S55. Schematics and pictures of the PEEK gel chamber and rod-shaped gel examples without and with film support (the latter was used for the actuation experiments).

To assemble the PEEK mold, a small amount of silicon grease was applied to the flat surfaces around the chamber openings on both sides of the mold. Subsequently, glass objective slides were attached and a commercial black foldback clip was used to hold the setup together. After a rubber seal was attached on top and the inner volume was purged with argon for 2 mins, the degassed gel precursor solution was loaded into each of the chambers. The precursor-loaded PEEK mold was then placed in an oven overnight at 60 °C. For the gels used in the actuation experiments, at the flat bottom of each chamber, a thin filter paper strip (cellulose filter) was fixated prior to the loading of the chamber with the precursor solution. In this way, the filter paper was successfully embedded as a support layer into the gel.

6 Actuation Experiments

To induce actuation of the gel samples, they were furnished with a solid support. Synthesis of the rod-shaped gels proceeded as previously described, except that at the lower bottom of each chamber a strip of filter paper was fixated before the PEEK mold was assembled, purged with Argon, and filled with the precursor solution. The cured gel was then carefully removed from the mold and the gel sample was cut into the desired dimensions (L×W×H) using a razor blade. Then the strip of filter paper --- now embedded into the gel --- was fixated with a tweezer. Next, the gel specimen was lowered to hang inside a glass cuvette (L × W × H = 50 × 14 × 50 mm) prior to addition of a 10 mM $[Bu_4N]^+[F]^-$ DMSO solution (10 mL).

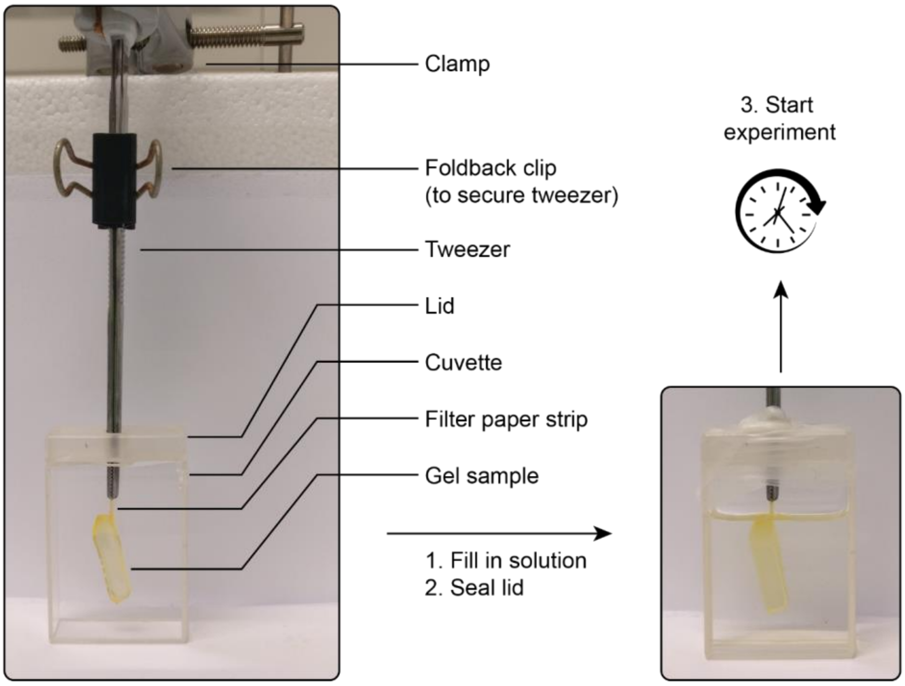

Figure S56. Experimental set-up for the actuation experiments.

Subsequently, the cuvette was sealed with a lid and parafilm to prevent water absorption of the hygroscopic solution. Pictures of the floating gel sample were taken from equal perspective and

distance at specific time points (0, 1, 2, 4 and 24 h). After 24 h, the solution was carefully replaced by a 0.8 M $[Bu_4N]^+[F]^-$ using a syringe. Pictures were taken again from equal perspective and distance at specific time points (0, 1, 2, 4 and 24 h). The evaluation of the pictures was carried out with Adobe Illustrator. The initial arm of the angle was aligned with the gel and the remaining paper strip that was held by the pair of tweezers. The terminal arm of the angle was aligned with the lower bent part of the gel sample. Both lines were extended to intersect and at this vertex the angle was measured on the monitor using a commercial protractor. The final plots show the change in angle ($\Delta = \delta_t - \delta_{t=0}$) vs. measurement time.

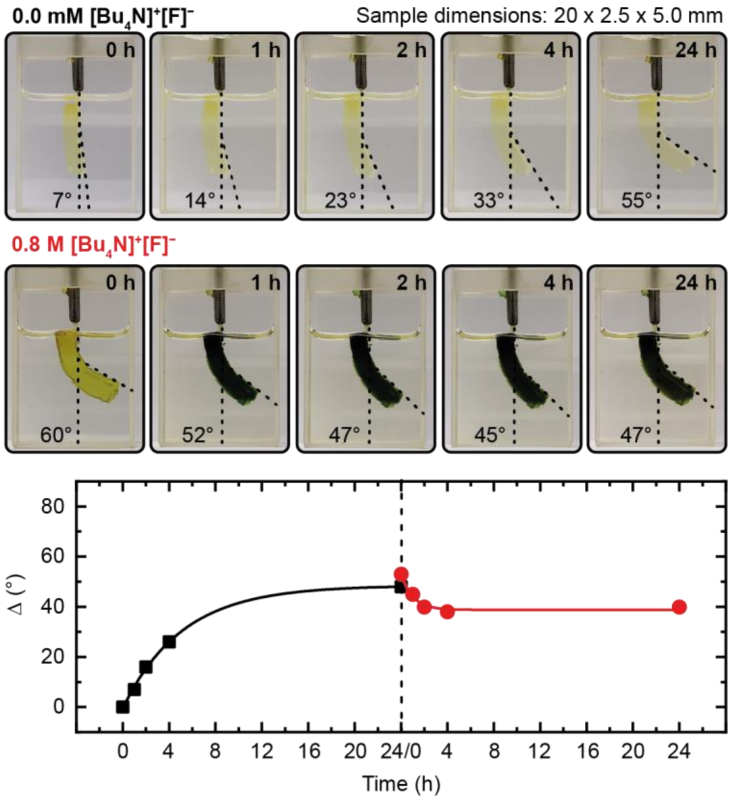

Figure S57. Actuation control experiment using **SQ1**-crosslinked gel (L×W×H = 20×2.5×5.0 mm, 5.0 mol% crosslinker) showing pictures of the bending specimen over time and the degree of bending D plotted vs. time. Lines are drawn manually to guide the eye.

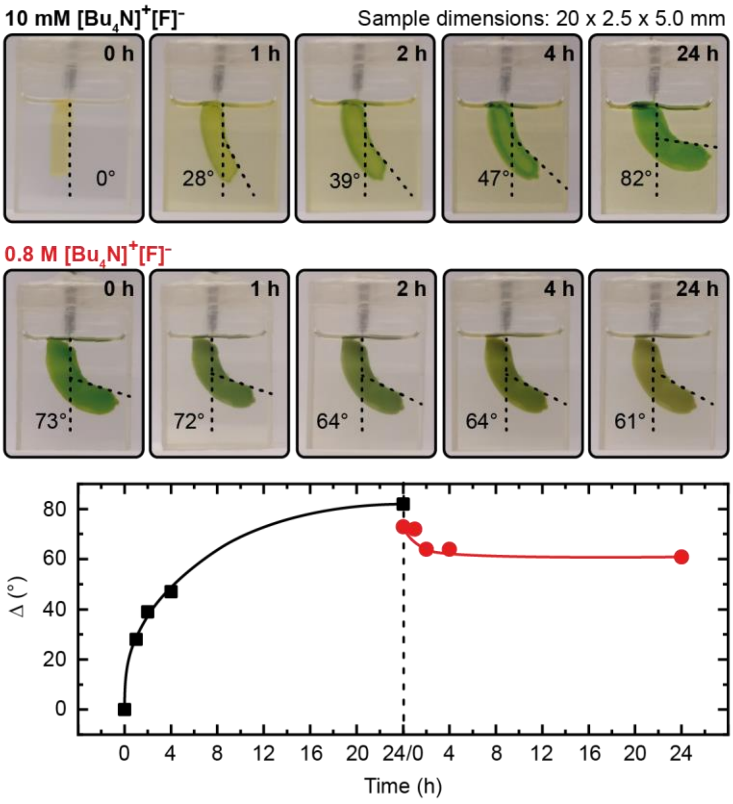

Figure S58. Actuation experiment using **SQ1**-crosslinked gel (L×W×H = 20×2.5×5.0 mm, 2.5 mol% crosslinker) showing pictures of the bending specimen over time and the degree of bending D plotted vs. time. Lines are drawn manually to guide the eye.

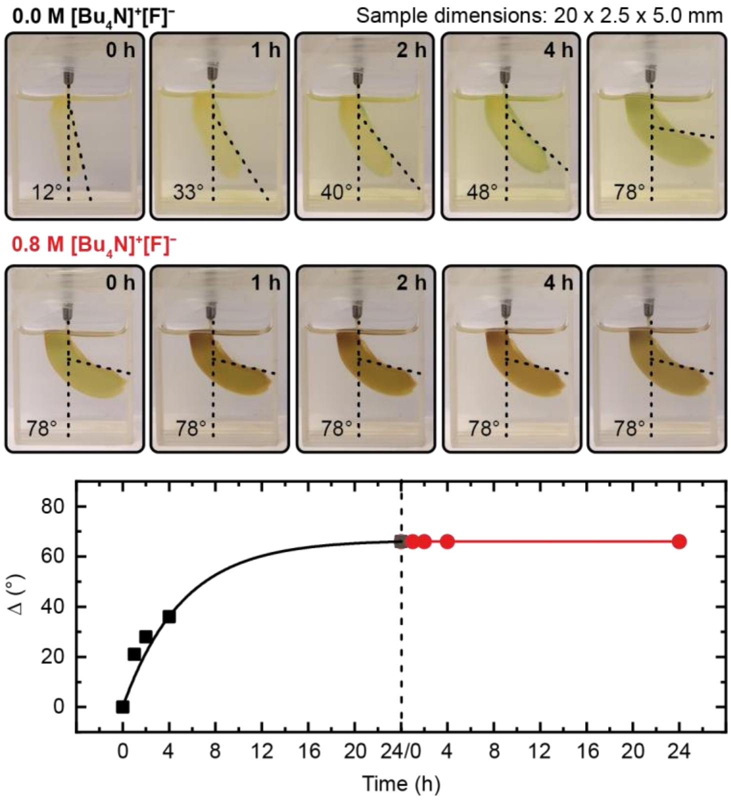

Figure S59. Actuation control experiment using **SQ1**-crosslinked gel (L×W×H = 20×2.5×5.0 mm, 2.5 mol% crosslinker) showing pictures of the bending specimen over time and the degree of bending D plotted vs. time. Lines are drawn manually to guide the eye.

7 References

1. Frassineti, C.; Ghelli, S.; Gans, P.; Sabatini, A.; Moruzzi, M. S.; Vacca, A., Nuclear Magnetic Resonance as a Tool for Determining Protonation Constants of Natural Polyprotic Bases in Solution. *Anal. Biochem.* **1995,** *231* (2), 374-382.
2. http://supramolecular.org, *BindFit Software Package*.
3. Hibbert, D. B.; Thordarson, P., The death of the Job plot, transparency, open science and online tools, uncertainty estimation methods and other developments in supramolecular chemistry data analysis. *Chem. Commun.* **2016** *52*, 12792-12805.
4. Richbourg, N. R.; Peppas, N. A., The swollen polymer network hypothesis: Quantitative models of hydrogel swelling, stiffness, and solute transport. *Prog. Polym. Sci.* **2020,** *105*, 101243.
5. Flory, P. J., *Principles of Polymer Chemistry*. Cornell University Press: Ithaca, NY: 1953.
6. Celik, T.; Orakdogen, N., Effect of charge density on water sorption and elasticity of stimuli-responsive poly(acrylamide–itaconic acid) and poly(N,N-dimethylacrylamide–itaconic acid) hydrogels: Comparison of experiment with theory. *J. Mater. Res.* **2013,** *28* (23), 3234-3244.
